# Tissue-specific isoforms of the single *C*. *elegans* Ryanodine receptor gene *unc-68* control specific functions

**DOI:** 10.1371/journal.pgen.1009102

**Published:** 2020-10-26

**Authors:** Filipe Marques, Saurabh Thapliyal, Avelino Javer, Priyanka Shrestha, André E. X. Brown, Dominique A. Glauser

**Affiliations:** 1 Department of Biology, University of Fribourg, Fribourg, Switzerland; 2 MRC London Institute of Medical Sciences, London, United Kingdom; 3 Institute of Clinical Sciences, Imperial College London, London, United Kingdom; Princeton, UNITED STATES

## Abstract

Ryanodine receptors (RyR) are essential regulators of cellular calcium homeostasis and signaling. Vertebrate genomes contain multiple RyR gene isoforms, expressed in different tissues and executing different functions. In contrast, invertebrate genomes contain a single RyR-encoding gene and it has long been proposed that different transcripts generated by alternative splicing may diversify their functions. Here, we analyze the expression and function of alternative exons in the *C*. *elegans* RyR gene *unc-68*. We show that specific isoform subsets are created via alternative promoters and via alternative splicing in *unc-68* Divergent Region 2 (DR2), which actually corresponds to a region of high sequence variability across vertebrate isoforms. The expression of specific *unc-68* alternative exons is enriched in different tissues, such as in body wall muscle, neurons and pharyngeal muscle. In order to infer the function of specific alternative promoters and alternative exons of *unc-68*, we selectively deleted them by CRISPR/Cas9 genome editing. We evaluated pharyngeal function, as well as locomotor function in swimming and crawling with high-content computer-assisted postural and behavioral analysis. Our data provide a comprehensive map of the pleiotropic impact of isoform-specific mutations and highlight that tissue-specific *unc-68* isoforms fulfill distinct functions. As a whole, our work clarifies how the *C*. *elegans* single RyR gene *unc-68* can fulfill multiple tasks through tissue-specific isoforms, and provide a solid foundation to further develop *C*. *elegans* as a model to study RyR channel functions and malfunctions.

## Author summary

The use of alternative exons allows a vast diversification of the isoform sequences encoded by single genes. The functional study of gene product isoforms *in vivo* using classical genetic approaches is challenging, notably because different isoforms may work in a partially redundant manner for many of their functions. Logically, research has progressed more rapidly in discovering the function of whole genes than that of specific isoforms. Using *C*. *elegans* model, we take advantage here of the recent development of efficient genome editing techniques to create alternative exon-specific molecular lesions and infer their specific roles *in vivo* by using a large set of phenotypic measures. We applied this approach to elucidate the role of alternative exons in the Ryanodine receptor (RyR) gene *unc-68*, which encodes the largest ion channel reported so far. Our results have implications for the understanding of the functional diversity of RyR channels across phyla and send an encouraging signal for the future application of genome editing to elucidate the functions of alternative exons in *C*. *elegans*.

## Introduction

Calcium is a prevalent second messenger controlling many cellular functions and playing a critical role in health and disease [1–3]. In excitable cells, intracellular calcium signals are used to couple stimuli with cell activity. Such activity could be e.g. neurotransmitter release by neurons or contraction in muscles. The Ryanodine Receptors (RyRs) are calcium-activated calcium channels expressed at the membrane of the endoplasmic/sarcoplasmic reticulum (ER/SR)[4]. One of their functions is to amplify intracellular calcium signals in the cytosol by mobilizing intracellular stores from the ER/SR, in a process called Calcium-Induced Calcium Release (CICR)[5]. RyR channels can profoundly impact the spatio-temporal pattern of calcium signals and modulate the degree of coupling between excitation and resulting activity. RyR channels are the largest channels reported to date. Each channel consists in the homotetrameric assembly of four RyR proteins. The channel pore is formed by the C-terminal parts of RyR proteins, while their N-terminal regions form a very large cytosolic structure able to dock many other proteins and small molecules to regulate channel opening [6, 7]. In mammals, three types of RyR proteins are found, RyR1, RyR2 and RyR3, each encoded by a separate gene. RyR1 is predominantly expressed in skeletal muscle, RyR2 in cardiac muscle and RyR3 in the nervous system. The three RyR proteins are close homologs, with about 65% sequence identity [8]. However, their sequences differ the most in three *divergent regions* named DR1, DR2 and DR3 [7, 9].

In human, RyR channels are implicated in many pathological states [10]. Mutations in RyR1 are associated with many muscle diseases, such as malignant hyperthermia (MH), exertional heat illness (EHI), central core disease (CCD), and late-onset axial myopathy (LOAM). Mutations in RyR2 are linked to catecholaminergic polymorphic ventricular tachycardia (CPVT) and arrhythmogenic right ventricular dysplasia type 2 (ARVD2)[11]. RyR2 also plays a role in heart failure independently of genetic mutations [12]. No genetic disease has been linked so far with mutations in RyR3. However, it has been proposed that RyR3 might play a role in Alzheimer disease [13]. Cell culture and animal models are crucial to study RyR function and malfunction [14]. As a powerful genetic model, allowing fast transgenesis or genome editing, cellular imaging *in vivo* and direct phenotypic recordings of muscular and neural functions, *C*. *elegans* has recently been established as a promising model to study the bases of RyR-linked disease. Like in all invertebrate examined so far [15], *C*. *elegans* genome encodes a single RyR channel gene, *unc-68 (uncoordinated-68)* [16]. Immunostaining analyses showed that UNC-68 is expressed in the body wall, pharyngeal, vulval, anal and sex muscles of *C*. *elegans* [17, 18]. In addition to those muscular tissues, reporter analyses have suggested that UNC-68 is also expressed in neurons [17], an observation confirmed by functional studies in neurons [19, 20]. Remarkably, while displaying impaired locomotion, neural regenerative potential and slowed pharyngeal pumping, null mutants of *unc-*68 are viable, which makes them very useful for genetic analyses. Baines and collaborators expressed *unc-68* variants containing mutations homolog to human mutations causing MH, CCD, EHI and LOAM, in an *unc-68* null background [21]. They recapitulated many critical features of corresponding human mutations in RyR1, including increased sensitivity to halothane and to caffeine, and a genetic dominance of those specific mutations. Fischer and collaborators created an optogenetic arrhythmia model based on *C*. *elegans* pharyngeal pumping [22]. They could impair this rhythmic function by introducing mutated UNC-68, containing a specific causal RyR2 mutation in CPVT and furthermore alleviate this phenotype with a benzothiazepine compound. Collectively, these studies demonstrate the potential of *C*. *elegans* for studying RyR channel function *in vivo* and for disease modelling.

It is not clear how functions similar to that of the three different RyR genes in mammals can be carried out by a single gene in *C*. *elegans*. One possibility is that different isoforms generated through alternative transcriptional start and/or splicing could be expressed in specific tissue and carry out specific functions. With this respect, it is noteworthy that the *C*. *elegans unc-68* gene includes many alternative exons and two likely transcription start sites (S1 Fig). Here, we show that specific isoform subsets are created via alternative transcription start site selection (exon 1.1 versus exon 1.2) and alternative splicing in the DR2 region of *unc-68* (exons 10, 12, and 13) and that they are differentially expressed in body wall muscle, neurons and pharyngeal muscle. Furthermore, by manipulating the endogenous alternative promoters and exons of *unc-68* with CRISPR/Cas9-mediated editing, we demonstrate their utility in modulating many biological functions such as pharyngeal pumping, swimming and crawling behaviors. Our data provide a comprehensive map of the pleiotropic impact of tissue-specific *unc-68* alternative exons, highlighting that they fulfill specific functions. As a whole, our work clarifies how the *C*. *elegans* single RyR gene *unc-68* can fulfill multiple tasks through tissue-specific isoforms, and provides a solid foundation to further develop *C*. *elegans* as a model to study RyR channel functions and malfunctions.

## Results

### Alternative promoters define tissue-specific expression of different isoforms of *unc-68*

**Fig 1:**
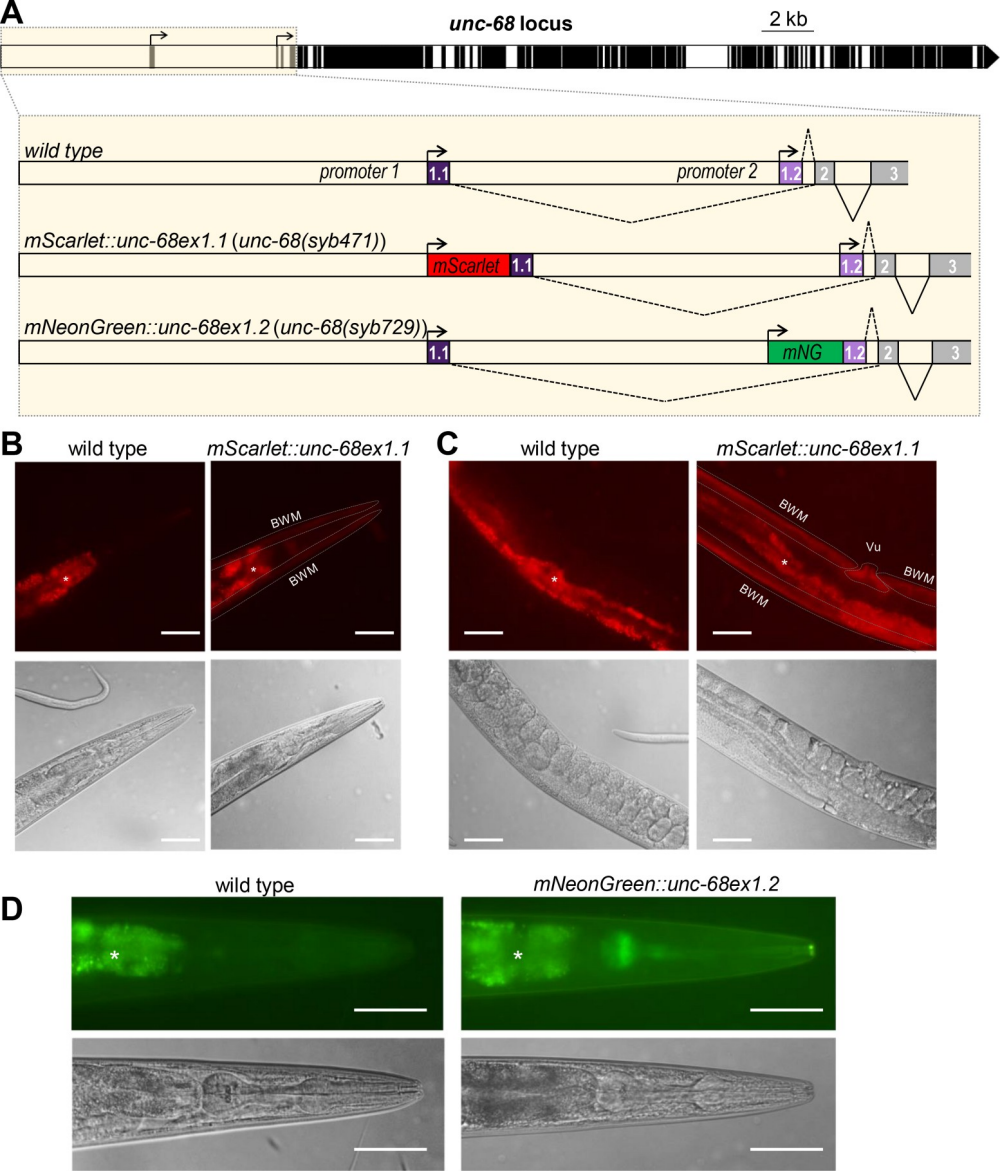
Alternative transcription analysis of *unc-68* through fluorescent reporter knock-ins. (A) Schematic of the *C*. *elegans unc-68* locus (exons in black, introns and promoters in white), highlighting alternative transcription start sites and location of fluorescent protein sequence knock-ins made by CRISPR/Cas9-mediated genome editing. mNG: mNeonGreen. (B, C, D) Representative fluorescence and DIC images taken in the head (B, D) and midbody (C) of wild type, *[mScarlet*::*unc-68ex1*.*1]* (B, C), *and [mNeonGreen*::*unc-68ex1*.*2]* (D) transgenic animals. BWM: Body wall muscle; Vu: Vulval muscle; *, autofluorescence background in the intestine. Scale bar: 50 μm.

Two alternative transcriptional start sites leading to the expression of two mutually exclusive first exons (exon 1.1 or exon 1.2) have been defined for *unc-68*, but nothing is known about isoform expression patterns. To address this question, we edited the *C*. *elegans* genome to specifically flag each isoform. Fluorescent UNC-68 isoforms were produced by fusing mScarlet or mNeonGreen coding sequence upstream of exon 1.1 and exon 1.2, respectively (Fig 1A). Overall, the fluorescent signal obtained using these (single copy) genomic knock-in reporters was relatively low, often comparable or weaker than that caused by the intestine autofluorescence. mScarlett::UNC-68(Ex1.1) fusion produced detectable signal in vulval and body wall muscle, but not in pharyngeal muscle (Fig 1B and 1C). In contrast, mNeonGreen::UNC-68(Ex1.2) signal was predominant in pharyngeal muscle, but barely detectable in body wall muscle (Fig 1D). For both transgenes, we sporadically observed extremely weak signals in neurons. While we cannot rule out that the inserted fluorescent protein sequences interfere with the regulation of *unc-68* expression, these data suggest that body wall muscle expresses predominantly exon 1.1-containing *unc-68* isoforms, that pharyngeal muscle expresses predominantly exon 1.2-containing *unc-68* isoforms, and that neurons may express both types.

**Fig 2:**
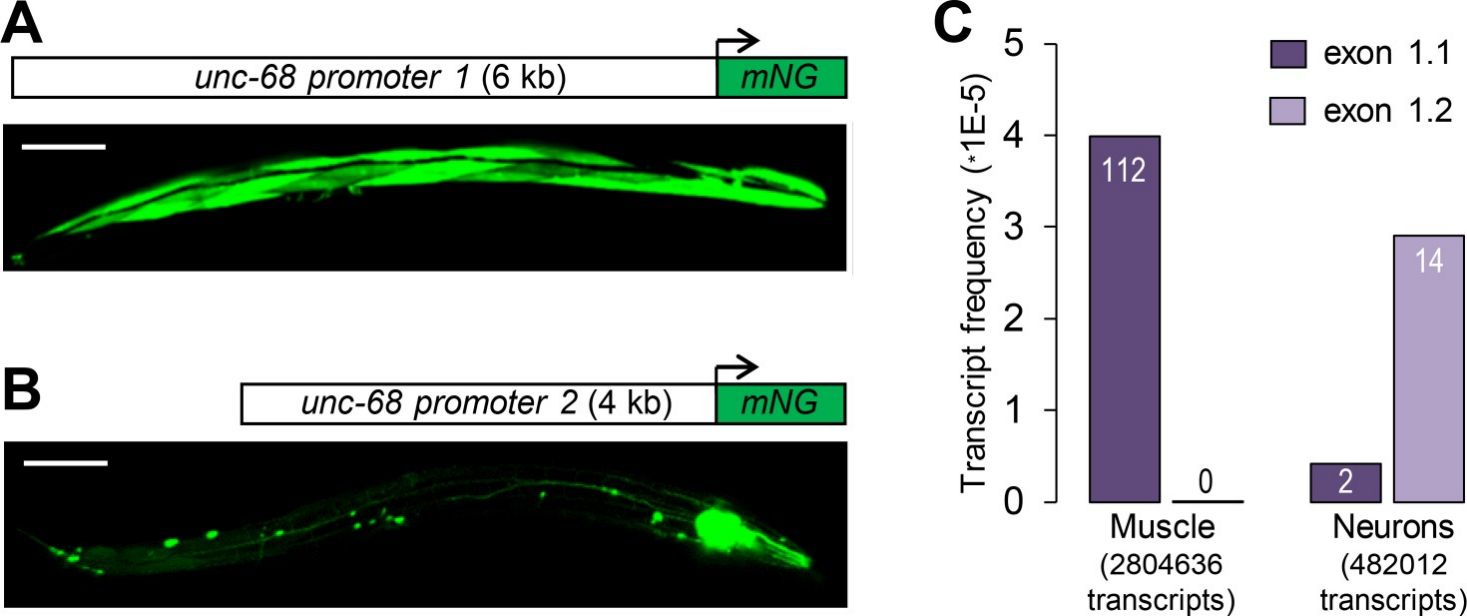
Alternative promoters and tissue-specific expression of *unc-68* exon 1.1 and exon 1.2. (A, B) Expression analysis using transcriptional reporters for *unc-68* promoter 1 (A) and 2 (B), respectively. At least three independent transgenic lines produced similar expression patterns. mNG: mNeonGreen. Scale bar: 100 μm. (C) Analysis of *unc-68* exon 1.1 and exon 1.2 inclusion in transcript pools enriched for *myo-3*-expressing muscle (mostly body wall muscle) and *rgef-1*-expressing neurons. Vertical axis indicates the fraction of analyzed transcripts accounted for by the corresponding *unc-68* transcript. Raw numbers of transcripts are indicated on the bars. The analysis was made from raw data published by Ma and collaborators [24].

In order to determine the role of putative regulatory regions lying upstream of each alternative first exon in driving tissue-specific expression, we created green fluorescent mNeonGreen transcriptional reporters for each region (promoter 1 and promoter 2, Fig 2A and 2B) and microinjected them in the gonad of wild type (N2) animals. The fluorescence signal in the resulting extrachromosomal array-carrying stable lines (Fig 2A and 2B) was markedly stronger than that produced by single copy reporter knock-in (Fig 1). This could be explained by the higher transgene copy number in the extrachromosomal array lines, as well as by the different sizes of the reporter proteins (~600 kDa for the knock-in fusions versus ~30 kDa for the fluorescent proteins alone). We found that promoter 1 drove strong expression in body wall muscles and more sporadic expression in some neurons, similar to previous observations with a β-galactosidase reporter (Fig 2A, [23]). In contrast, expression driven by promoter 2 was restricted to neurons and pharyngeal muscle (Fig 2B).

In order to address whether promoter 1 and promoter 2 drive expression in the same or a different subset of neurons, we created transgenic animals co-injected with two constructs: *[unc-68p1*::*NLS*::*mScarlet]* and *[unc-68p2*::*mNeonGreen]*. We could detect neurons expressing only the red marker, neurons expressing only the green marker, and neurons expressing both markers (S2 Fig). Neurons in the latter category were rarer and located solely in the tail region. We cannot rule out that the presence of single-color neurons could be due to variable expression from the extrachromosomal array. Taken together, the results of this dual marker analysis suggest that the two promoters drive transcription in at least partially overlapping neuron sets.

To confirm these reporter-based observations, we analyzed tissue-enriched transcriptomic data obtained in neurons and body wall muscles by Ma and collaborators using the trans-splicing-based RNA tagging (SRT) method [24]. One hundred percent of the *unc-68* transcripts recovered in the body wall muscle-enriched mRNA pool contained exon 1.1 (n = 112. Fig 2C). In contrast, the neuron-enriched mRNA pool contained both exon 1.1 and 1.2 isoforms (12% and 88%, respectively, n = 16).

Collectively, these data indicate that neurons and different muscle types express different *unc-68* isoforms varying in their first exon and that regulatory elements located within promoter 1 and promoter 2 contribute to control tissue-specific expression.

### Alternative splicing diversifies the sequence of the UNC-68 DR2 domain

**Fig 3:**
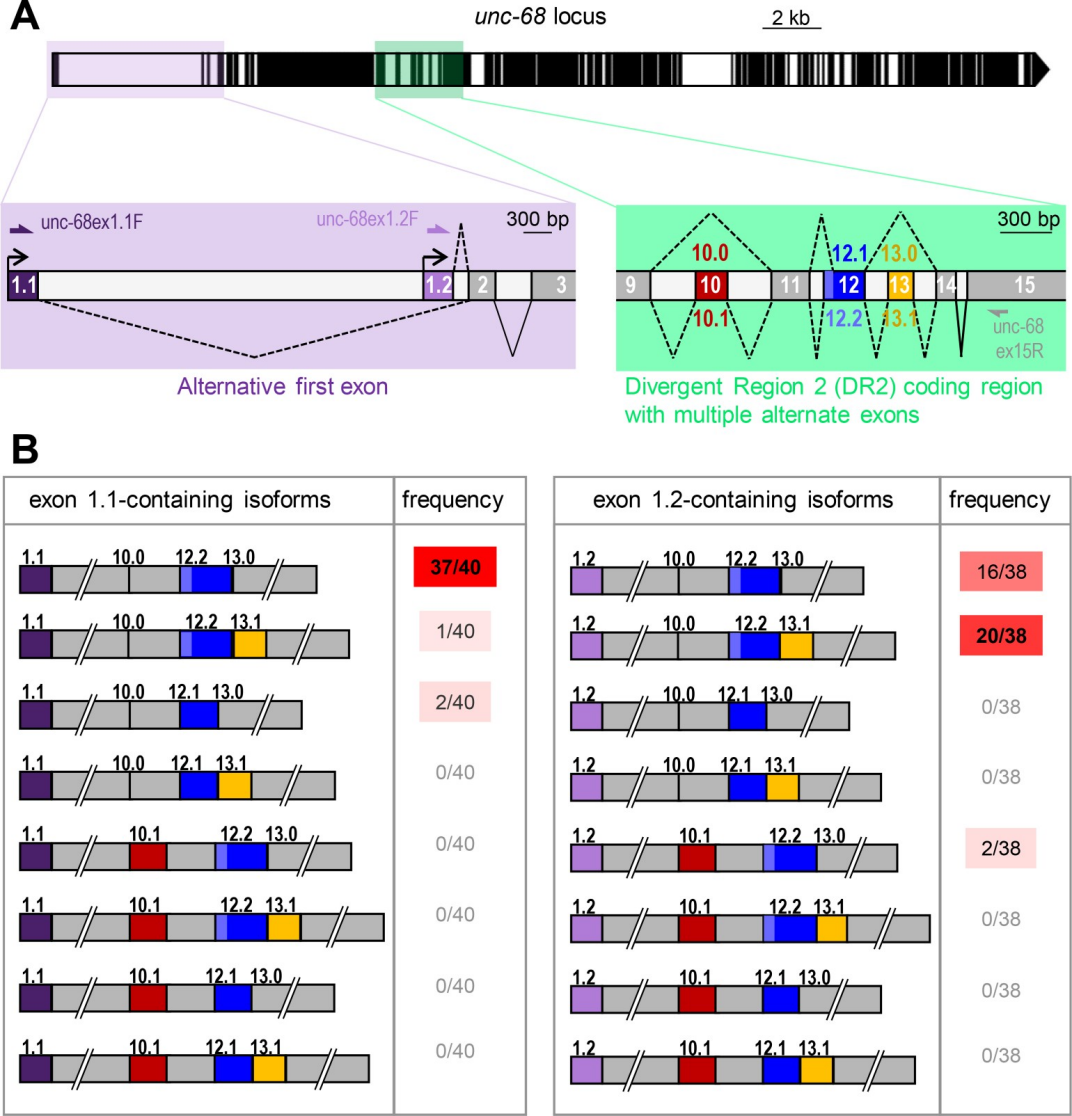
Combinations of *unc-68* alternative exons. (A) Schematic of the *unc-68* locus as in Fig 1, with close-up views of the alternative transcription start region (lavender) and of the alternative splicing hot spot in the DR2 coding region (green). The positions of primers used for the alternative exon combination analysis are indicated. (B) Schematic representation and frequency of each of the 16 possible isoforms resulting from the combination of alternative exons 1,10, 12 and 13. Only 6 isoforms were detected.

Marked sequence variations in the DR2 region are linked to functional differences across the three mammalian RyR channels. We thus wondered whether alternative splicing within the corresponding region in the *unc-68* gene of *C*. *elegans* could diversify its sequence. While a recent *unc-68* gene structure model does not report alternative splicing in this region (Wormbase WS274, March 2020, S1 Fig), earlier models did so (Wormbase version WS260). We empirically confirmed the existence of an alternative splicing hotspot in the region covering exons 10 to 13 of *unc-68* (see sequencing analysis results in the next paragraph) and corresponding to DR2 in mammals (see protein sequence alignment in S3 Fig). As schematically depicted in Fig 3A, exon 10 can be either skipped (10.0) or included (10.1), exon 12 can exist in a short (12.1) or long form (12.2) via alternative splice donor sites, and exon 13 can be either skipped (13.0) or included (13.1). The definition of these exons at a nucleotide-level resolution is available in S4 Fig. Interestingly, we noticed a strong sequence conservation across exons and splice acceptor/donor sites in this gene region within the *Caenorhabditis* genus despite a phylogenetic divergence more than 20 million years ago (S4 Fig). These observations about DR2 sequence diversification through alternative splicing and the sequence conservation within the *Caenorhabditis* genus support the hypothesis that different isoforms created from the single *C*. *elegans unc-68* gene may fulfill different roles, similar to those of the different RyR genes.

### Expression of specific *unc-68* alternative exon combinations

Next, we wondered whether alternative splicing within the DR2 region of *unc-68* could also be linked to tissue-specific alternative transcription start site selection. Thus, we compared the alternative exon composition in the DR2 region (exons 10, 12 and 13) between exon 1.1 and exon 1.2-containing transcripts. To do so, we PCR-amplified each transcript type from a mixed stage worm cDNA library using forward primers specific to either exon 1.1 or 1.2 and a reverse primer in the constitutive exon 15 (Fig 3A). Cloned PCR products were then sequenced (40 clones with exon 1.1 and 38 with exon 1.2). Over 78 clones analyzed, we detected each of the previously reported alternative exon configurations in the DR2 region, namely Ex10.0 (exon 10 skipping), Ex10.1 (exon 10 inclusion), Ex12.1 (short version of exon 12), Ex12.2 (long version of exon 12), Ex13.0 (exon 13 skipping) and Ex13.1 (exon 13 inclusion). However, out of 16 possible combinations (2 *alternative promoter* x 2 *alternative exon 10* x 2 *alternative exon 12* x 2 *alternative exon 13*), we detected only 6 isoforms (Fig 3B). The vast majority of the exon 1.1-containing clones (38/40) were of the 1.1/10.0/12.2/13.0 type (Fig 3B). Thus, the predominant *unc-68* body wall muscle isoform includes the long version of exon 12 but does not include exons 10 and 13 (this corresponds to the gene model in wormbase WS274). In contrast, the majority of the exon 1.2-containing clones (20/38) were of the 1.2/10.0/12.2/13.1 type, hence including exon 13. The second most frequent isoform was 1.2/10.0/12.2/13.0. Overall, the inclusion of exon 10 or of the short exon 12 (12.1) were the least frequent events (each observed in only 2/78 clones). Care should however be taken in interpreting the results about the least frequent alternative exon combinations, as we cannot totally rule out the creation of artifactual chimeric PCR products in the course of the analysis [25]. Collectively, these data indicate that sequence variations occur in the DR2 region of UNC-68 through alternative splicing and that specific DR2 variations are associated with tissue-specific alternative transcription start.

### Tissue-specific expression of *unc-68* alternative exon 13

**Fig 4:**
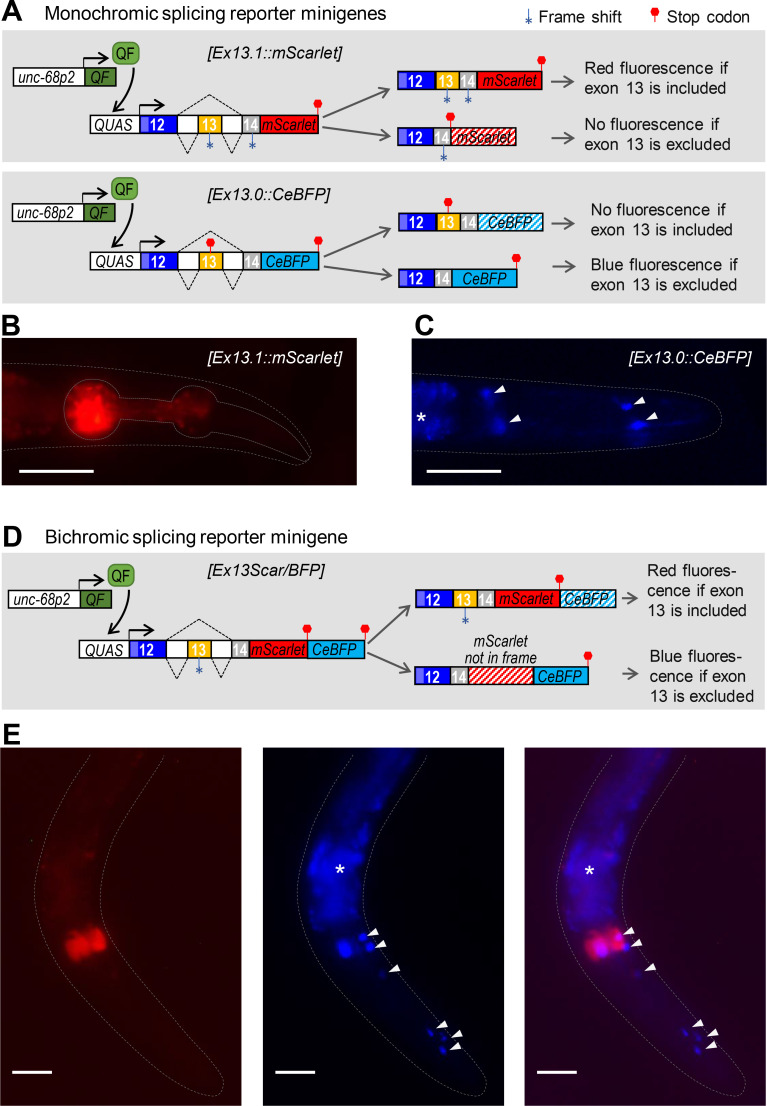
Expression of *unc-68* alternative exon 13 in specific tissues. The expression of *unc-68* exon 13 was analyzed using alternative splicing reporter minigenes. (A, D) Schematic of the monochromic (A) and bichromic (D) *unc-68* exon 13 alternative splicing reporter minigenes and of the expression system. The Q-system (including *unc-68p2*::*QF* and *QUAS*::*’reporter’* transgenes) was used to drive the transcription of each minigene into pharyngeal muscle and neurons. For each of them, exon 13 inclusion yields transcripts with an in frame mScarlet sequence, and its exclusion yields transcripts with an in frame CeBFP sequence. (B, C, E) Representative fluorescence images of transgenic animals expressing the monochromic (B, C) and bichromic (E) *unc-68* exon 13 alternative splicing reporters. Arrow heads: unidentified neuronal cells. *, autofluorescence background in the intestine. Scale bar: 50 μm.

The fact that exon 13 inclusion was strongly enriched in exon 1.2-containing transcripts (Fig 3), which are synthesized only in specific tissues, suggests that exon 13 inclusion could be a tissue-specific event. To examine this possibility, we developed two parallel alternative splicing reporter minigene strategies (Fig 4A and 4D). As detailed below, these minigenes were designed to report the inclusion or exclusion of exon 13, via the cell-autonomous expression of fluorescent proteins, using previously described strategies [26, 27]. Our initial goal was to express these reporters in all UNC-68-expressing tissues using both promoter 1 and promoter 2 to drive the transgene expression. Unfortunately, we faced transgene toxicity issues when these minigenes where expressed in body wall muscle (see details in the Methods section) and the exon 13 inclusion analysis presented below is therefore limited to pharyngeal muscle and neurons using *unc-68* promoter 2.

In the first approach (Fig 4A), we created two monochromic reporters where the *unc-68* genomic sequence from exons 12 to 14 was fused upstream of either mScarlet or CeBFP coding sequence. In the *[Ex13*.*1*::*mScarlet]* reporter, exon 13 inclusion produces an in frame transcript and a red UNC-68(ex12/ex13/ex14)::mScarlet protein, but its exclusion creates a frameshift and a premature stop. Red signal was detected only in pharyngeal muscle (Fig 4B), suggesting that exon 13 inclusion takes place in this tissue. In the *[Ex13*.*0*::*CeBFP]* reporter, exon 13 inclusion creates a frameshift and a premature stop, but its exclusion produces an in frame transcript coding for a blue UNC-68(ex12/ex14)::CeBFP protein. Blue signal was detected only in neurons, suggesting exon 13 exclusion.

In the second approach, we used a bichromic *[Ex13Scar/BFP]* reporter in which exon 13 inclusion creates a red UNC-68(ex12/ex13/ex14)::mScarlet protein, whereas exon 13 exclusion leads to a frame shift over the *mScarlet* sequence (nevertheless devoid of stop codons) and the production of a blue UNC-68(ex12/ex14)::out-of-frame mScarlet::CeBFP reporter protein. We observed red signal in the pharyngeal muscle (indicating exon 13 inclusion, Fig 4E) and blue signal mostly in neurons (indicating exon 13 exclusion, Fig 4E).

Collectively, the results of our two alternative splicing reporter minigene approaches converge to suggest that exon 13 is included in pharyngeal muscle *unc-68* isoforms and excluded in neuronal isoforms.

### *unc-68* alternative promoter and first exons differentially regulate animal behaviors

**Fig 5:**
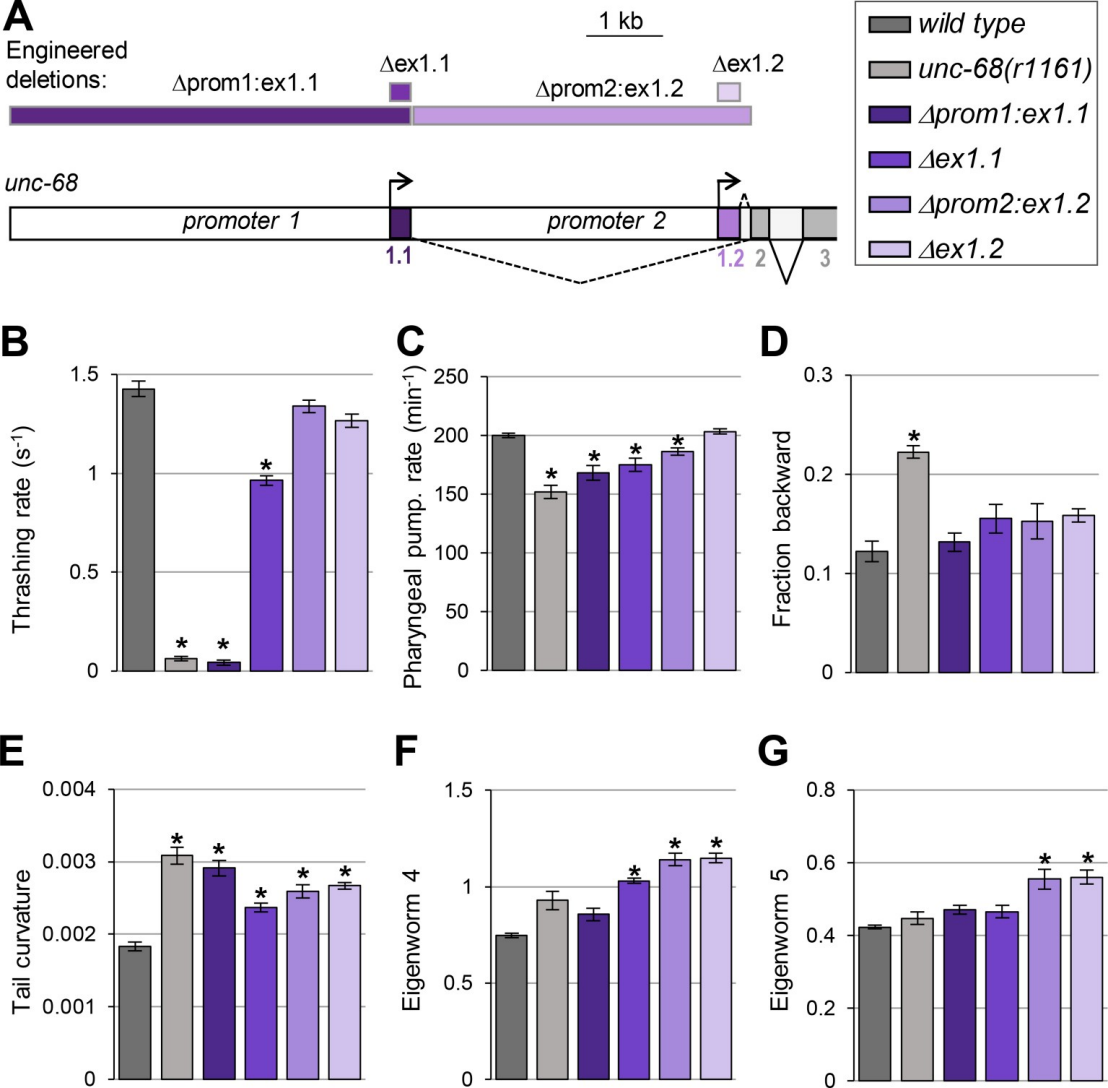
The loss of *unc-68* alternative first exons and promoters impact behavior. (A) Schematic of the 5’ region of the *unc-68* locus and location of the engineered deletions produced by CRISPR/Cas9-mediated genome editing (rectangles at the top of the panel). (B-G) Phenotypic analyses comparing wild type (N2), an *unc-68* null mutant (*unc-68(r1161)*) and the indicated engineered mutants. Results as means (+/- s.e.m). *n*≥32 animals (B); *n*≥38 animals (C); *n* = 5 plates (each with at least 10 tracked animals (D-G)). *, *p* < .01 versus wild type by Dunnett’s tests.

Our next goal was to examine the functional role played by *unc-68* alternative promoter and first exons. To that end, we compared the phenotype of wild-type, *unc-68(r1161)* null mutants, and mutant lines that we engineered to lack specific alternative exons and/or promoters (Fig 5A). We created four lines:

1. *Δprom1*:*ex1*.*1* line lacking both ex1.1 and promoter 1. This line can only produce ex1.2-containing isoforms under the control of promoter 2.
2. *Δex1*.*1* line lacking only ex1.1. This line can only produce ex1.2-containing isoforms, but retains regulatory elements in promoter 1 and 2 regions, each of which may potentially affect transcription initiation upstream of ex1.2.
3. *Δprom2*:*ex1*.*2* line lacking both ex1.2 and promoter 2, where ex1.1 is directly fused to constitutive ex2. This line can only produce ex1.1-containing isoforms under the control of promoter 1.
4. *Δex1*.*2* line lacking only ex1.2. This line can only produce ex1.1-containing isoforms, but retains regulatory elements in promoter 1 and 2 regions, each of which may potentially affect transcription initiation upstream of ex1.1.

We analyzed phenotypes previously reported to be altered in *unc-68* null mutants, such as swimming and pharyngeal pumping behavior, as well as postural and locomotion phenotypes that we quantified with high-content computer-assisted behavior analysis.

#### Swimming behavior analysis

Normal *C*. *elegans* motility while swimming requires an intact *unc-68* gene [16], and UNC-68 expression both in neurons and in body wall muscle affects locomotion [19]. This process may therefore implicate multiple tissue-specific exons and promoters. We found that *Δprom1*:*ex1*.*1* animals had a severe swimming phenotype, with a marked reduction in thrashing rate undistinguishable from that in null mutants (Fig 5B). *Δex1*.*1* animals were also defective, but with an intermediate phenotype between that of wild type and that of *Δprom1*:*ex1*.*1* animals. These results show that exon 1.1-containing isoforms expressed under the control of promoter 1 are essential for normal swimming. In contrast, *Δprom2*:*ex1*.*2* or *Δex1*.*2* animals almost swum like wild type, suggesting that exon-1.2-containing isoforms are normally unnecessary for swimming. Furthermore, the fact that swimming is only partially affected in *Δex1*.*1* animals suggests that exon 1.2-containing isoforms may partially suffice to maintain *unc-68* function, provided that regulatory elements in promoter 1 are present.

#### Pharyngeal pumping analysis

Pharyngeal pumping in *C*. *elegans* is controlled by *unc-68* and depends on the intact function of pharyngeal muscle cells as well as on inputs from the nervous system [28]. This process may therefore implicate multiple isoforms of *unc-68*. Similar to previous findings [17, 22], we found that loss of *unc-68* significantly reduced the rate of spontaneous pharyngeal pumping (~24% reduction, Fig 5C). We observed a similar effect in *Δprom1*:*ex1*.*1* animals (not statistically different from *unc-68* null) and a slightly less pronounced reduction in *Δex1*.*1* animals (statistically different from *unc-68* null). Pharyngeal pumping was normal in *Δex1*.*2* animals, but significantly reduced in *Δprom2*:*ex1*.*2* animals. Collectively, these data suggest (i) that exon 1, promoter 1 and promoter 2 are all important for *unc-68-*mediated pharyngeal pumping control, (ii) that exon 1.2 is dispensable, but (iii) that exon 1.2 can partially compensate for the lack of exon 1.1 if promoters 1 and 2 are intact.

#### Locomotion and postural analysis in crawling animals

To deepen our understanding of the functional role of alternative first exons and promoters in *unc-68*, we expanded the phenotypic characterization of *unc-68* mutants using high-content computer-assisted crawling behavior analysis [29, 30]. We found that, compared to wild type, *unc-68* null animals spent more time in backward locomotion mode (Fig 5D) and displayed an increased tail curvature (Fig 5E).

In contrast to *unc-68* null mutants, none of the promoter and first exon-specific deletion mutants significantly increased the time spent in backward locomotion mode (Fig 5D). This suggests that *unc-68* isoforms containing different first exons, and expressed via different alternative promoters, function redundantly to regulate spontaneous backward movements.

Regarding the animal posture, we found that *Δprom1*:*ex1*.*1*, *Δex1*.*1*, *Δprom2*:*ex1*.*2* and *Δex1*.*2* mutants all displayed an increased tail curvature (Fig 5E). This effect was qualitatively similar to that in *unc-68* null mutants, but overall its magnitude seemed less pronounced in isoform-specific mutants. This suggests that *unc-68* controls this peculiar postural phenotype via exon 1.1 and exon 1.2-containing isoforms, which work in an only partially redundant manner. Still regarding animal posture, we found that the contributions of eigenworms 4 and 5 to the overall curvature pattern repertoire were overrepresented for some mutants, while it was not the case for *unc-68* null mutants (Fig 5F and 5G). These observations indicate that specific aspects of animal posture during crawling behavior are affected when specific *unc-68* isoforms are mutated, but not when all isoforms are affected at the same time. Taken together, these results indicate that specific *unc-68* isoforms differentially control specific postural parameters.

### Alternative exons affecting UNC-68 DR2 sequence regulate specific animal behaviors

**Fig 6:**
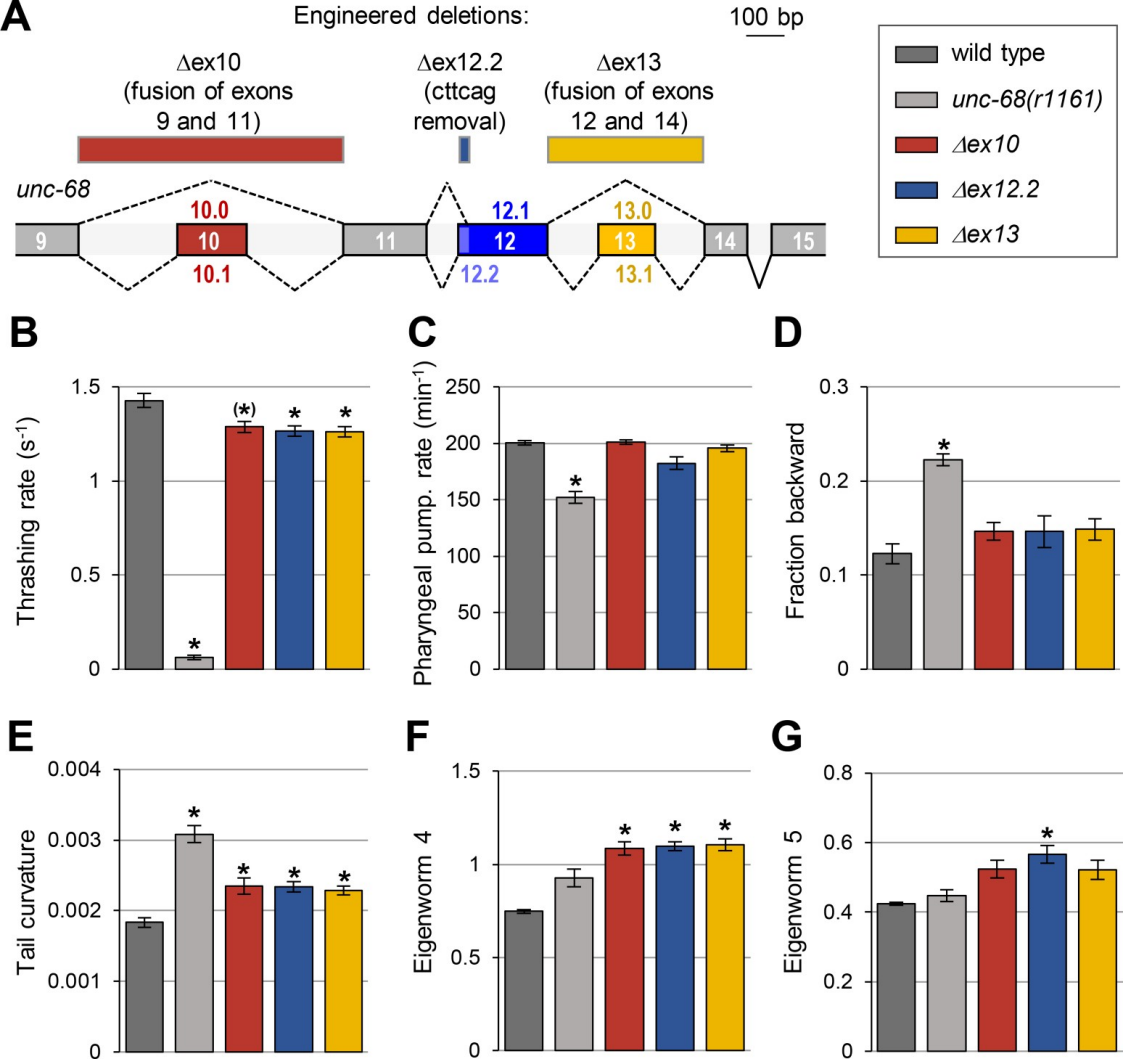
Alternative exons coding for the DR2 region of UNC-68 have specific roles. (A) Schematic of the DR2 coding region in the *unc-68 genomic* locus and location of the engineered deletions produced by CRISPR/Cas9-mediated genome editing (rectangles at the top of the panel). (B-G) Phenotypic analyses comparing wild type (N2), an *unc-68* null mutant (*unc-68(r1161)*) and the indicated engineered mutants. Results as means (+/- s.e.m). *n*≥38 animals (B); *n* = 30 animals (C); *n* = 5 plates (each with at least 10 tracked animals (D-G). *, *p* < .01 and (*), *p* < .05 versus wild type by Dunnett’s tests.

To examine the biological role of alternative splicing in the region coding for the DR2 domain of UNC-68, we performed a similar phenotypic characterization in three engineered deletion mutant lines (Fig 6A):

1. *Δex10* line, in which exons 9 and 11 are joined to eliminate the possibility of creating isoforms containing exon 10.
2. *Δex12*.*2* line, in which the 6 nucleotides specific to the long exon 12.2 are deleted leaving only the possibility to produce isoforms containing exon 12.1.
3. *Δex13* line, in which exons 12 and 14 are joined to eliminate the possibility of creating isoforms containing exon 13.

#### Swimming behavior analysis

We observed a significant reduction in the thrashing rate in *Δex10*, *Δex12*.*2* and *Δex13* animals (Fig 6B and S5 Fig). The magnitude of this effect was however very low in comparison to that caused by a full *unc-68* knock out, indicating that these alternate exons are only partly required to control worm swimming.

#### Pharyngeal pumping analysis

Unlike *unc-68* null mutants, none of the DR2 alternative exon mutants displayed a significant reduction in pharyngeal pumping (Fig 6C). These results indicate that none of alternative exon 10, 12.2 and 13 is required for this function.

#### Locomotion and postural analysis in crawling animals

First, regarding the locomotion mode, none of the DR2 alternative exon mutants recapitulated the increased time spent in backward locomotion found in the *unc-68* null mutants (Fig 6D), suggesting that exon 10, 12.2 and 13 are dispensable to regulate this behavior. Second, we found that DR2 alternative exon deletions modified the animal posture with increased tail curvature and eigenworm 4 contribution in *Δex10*, *Δex12*.*2* and *Δex13* mutants (Figs 6E, 6F and S5) and, additionally, an increased eigenworm 5 contribution in *Δex12*.*2* mutants (Fig 6G). These results indicate that UNC-68 isoforms diverging in the DR2 domain, control specific aspects of animal posture during crawling behavior.

## Discussion

Ryanodine receptors play important biological roles and are broadly conserved in vertebrates and invertebrates. In vertebrates they are encoded by two or more genes, with tissue-specific expression and function, whereas in invertebrates only one gene is found. Based on the existence of multiple invertebrate transcript isoforms generated via alternative splicing and on differential expression patterns, it has been repeatedly speculated that these isoforms may diversify Ryanodine receptors’ function (see [15] for a review). Using genome editing, we provide here, to our knowledge, the first evidence for a causal relationship between alternative exon usage and specific Ryanodine receptor-dependent functions in different tissues *in vivo*.

### Functional redundancy and diversity among *unc-68* isoforms

**Fig 7:**
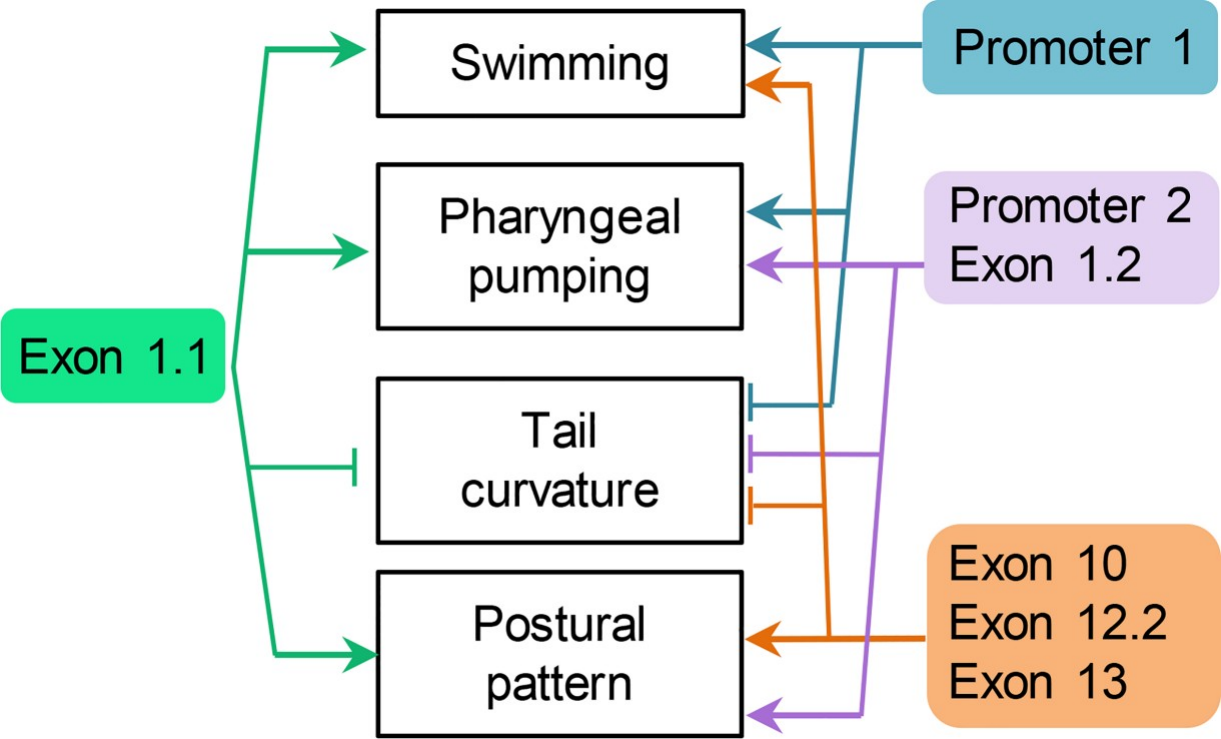
Task distribution among *unc-68* alternative promoters and exons. Schematic view summarizing the connections between specific phenotypic traits and *unc-68* alternative exons and promoters.

Our phenotypic analysis in *unc-68* engineered mutants sheds light on the role played by the diversity of *unc-68* isoforms created by the use of alternative exons and promoters (see schematic summary in Fig 7). The fact that the phenotypic impact of the null mutation markedly diverged from that of most alternative exon/promoter mutations, highlight that these alternative elements are non-essential for many functions and that remaining *unc-68* isoforms at least partly compensate for most phenotypes. In a few instances however, like for swimming, we found that the joint deletion of promoter 1 and exon1.1 was as severe as the full knock out. These observations indicate that, in that case, the ex1.2-containing isoforms expressed under the control of promoter 2 are not sufficient to compensate for the lack of ex1.1 and promoter 1. This result is corroborated by previous findings where a cosmid containing the full *unc-68* coding sequence, but lacking most of promoter 1, failed to rescue the swimming defect in *unc-68* knock out animals [17]. We also found specific phenotypes in alternative exon mutants that were not present in the null mutants (like the postural phenotype in the DR2 region mutants). A possible explanation, would be that different isoforms have opposite impacts on this phenotype, and that this regulation is only apparent when isoform expression is imbalanced. As a whole, our results suggest that the rich repertoire of *unc-68* isoforms is important to fine-tune many biological functions. For some functions, only specific isoforms are required, and for some others, different isoforms may function redundantly.

### Tissue-specific alternative transcription initiation site selection in *unc-68*

Previous studies have indicated that *unc-68* is expressed and/or plays a role in body wall muscle, pharyngeal muscle and neurons. To our knowledge, researchers focused so far on characterizing exon 1.1-containing *unc-68* isoforms. Our genome editing approach confirms past results and complements them with information on exon 1.2-containing isoforms. Furthermore, our transcriptional reporter analysis fusing promoter 1 or promoter 2 directly upstream of a fluorescent protein indicates that regulatory elements located in each of them are sufficient to drive tissue-specific expression. Consistent with previous LacZ reporter analysis [23], we found that promoter 1 was sufficient to drive expression in the body wall muscle and some neurons, while promoter 2 (corresponding to intron 1 in the exon 1.1-containing pre-messenger RNA) was sufficient to drive expression in the pharyngeal muscle, as well as some neurons. Based on our expression and phenotypic analysis results, it seems likely that regulatory elements in promoter 1 may not only control the transcription of exon 1.1 isoforms, but also regulate the expression of exon1.2 isoforms. Indeed, the phenotype observed in *Δpromoter1*:*ex1*.*1* mutant was in several instances stronger than that in *Δex1*.*1* mutants. In contrast, we found less evidence that regulatory elements within promoter 2 play a role to regulate exon 1.1-containing isoform. The phenotype in *Δpromoter2*:*ex1*.*2* was more severe than that in *Δex1*.*2* mutants only in the case of pharyngeal pumping. Since promoter 2 includes essential elements to drive expression in pharyngeal muscle, they could potentially work as downstream enhancers to regulate initiation from exon 1.1, at least in the absence of exon 1.2. It is important to keep in mind also that promoter 2 spans over intron 1 of exon 1.1-containing transcripts, and that the implicated regulatory element could work both pre- and post-transcriptionally. Obtaining a deeper view of the regulatory elements determining tissue-specific transcriptional start site selection and their functional consequences will require additional experiments, e.g. by editing the genome to produce more extensive and fine-grained deletions and using tissue-specific expression of selected isoforms.

### Role of alternative splicing in the *unc-68* region coding for the DR2

The divergent region 2 (DR2) in mammals is believed to contribute to the functional differences across the different RyR gene products. This domain was notably proposed to play a role in modulating excitation-contraction coupling in skeletal muscle, potentially by affecting the interaction between RyR and L-type calcium channels [31, 32]. Recent structural analyses suggest that the DR2 region is mostly unstructured [33]. We found that alternative splicing in *unc-68* exon 10, 12 and 13, diversifies the UNC-68 sequence in the corresponding domain. A protein sequence alignment comparing human and *C*. *elegans* reveals a much lower conservation in the DR2 domain, as compared to surrounding sequences (S3 Fig). We could not hence discern a clear relationship between specific human gene isoforms and specific *C*. *elegans* transcript isoforms. In particular, the DR2 region of *C*. *elegans* appears longer than that in human RyRs, and we could not delineate clearly homologous regions. Given the phylogenetic history of the RyR genes and their numbers, functional diversification most likely occurred independently, but converged to target a specific RyR protein region that is not strongly constrained in its structure.

Based on the results of our expression and functional analyses, we conclude that all three alternative exons in the DR2 region modulate UNC-68 function and, in the case of exon 13 at least, are enriched in specific tissues. Exon 13 inclusion hence appears mostly as a pharyngeal muscle-specific event. This conclusion is directly supported by splicing reporter minigene expression and indirectly supported by cDNA sequencing analysis. Indeed, we noted (i) a relative enrichment of exon 13 in pharynx-enriched exon 1.2-containing transcripts and (ii) a parallel exon 13 depletion in exon 1.1-containing isoforms, which are most likely contributed by the abundant body wall muscle tissue in our mRNA sequencing analysis. However, our results do not rule out that exon 13 may be included and have functional roles in additional tissues. Of note, the impairments observed in *Δex13* animals are not easily explained if exon 13 was acting solely in the pharyngeal muscle. It is also intriguing that animals lacking either exon 10 or 13, display very similar phenotypes, because we never detected them together in a unique transcript. It is possible that exons 10 and 13 are each required in a single isoform containing both of them, but expressed at low levels (and therefore not detected so far). Alternatively, they may similarly impact the DR2 domain function in separate, non-redundantly functioning isoforms. A third possibility could implicate genome editing off-target effects that could have occurred when generating the mutant lines. We however consider this latter possibility as relatively unlikely because (i) unrelated single-guide RNAs were used to generate each mutation and (ii) the phenotypic effects were maintained despite repeated outcrossing.

Many additional questions are now open regarding the function of the diversified DR2 region in UNC-68, as well as at other sites within the protein. Indeed, the most recent gene model indicates the existence of five further alternative exons in *unc-68* (S1 Fig). Our work serves as a proof of feasibility for a functional analysis of alternative exons *in vivo* via genome editing, and opens the road for additional structure/function studies addressing the diverse biological roles of UNC-68 isoforms.

## Conclusion

Altogether, our results show (i) that the alternative transcription start site selection and alternative splicing in the DR2 region define the expression of specific UNC-68 isoform sets in distinct tissues and (ii) that this regulation is essential to properly orchestrate the multiple specific biological functions carried out by these isoforms. Our findings clarify how the single *C*. *elegans* Ryanodine receptor gene may achieve functions carried out by several genes in mammals, and will be essential to implement further Ryanodine receptor studies in *C*. *elegans*, including promising disease modeling, for which understanding the endogenous activity and expression of specific isoforms is essential.

## Methods

### Worm strains

The strains used in this study were the following: N2, TR2171 *unc-68(r1161)*, DAG356 *domIs355 [mec-3p*::*QF*, *mec-4p*::*QS*, *QUAS*::*CoChR*::*GFP*, *unc122p*::*RFP]*, DAG634 *domEx634 [unc-68p2*::*mNeonGreen*::*unc-54 3’UTR]*, DAG635 *domEx635[unc-68p2*::*mNeonGreen*::*unc-54 3’UTR]*, DAG636 *domEx636[unc-68p1*::*mNeonGreen*::*unc-54 3’UTR]*, DAG637 *domEx637[unc-68p1*::*mNeonGreen*::*unc-54 3’UTR]*, PHX214 *unc-68(syb214)*, PHX215 *unc-68(syb215)*, PHX216 *unc-68(syb216)*, PHX217 *unc-68(syb217)*, PHX218 *unc-68(syb218)*, PHX219 *unc-68(syb219)*;PHX220 *unc-68(syb220)*, PHX471 *unc-68(syb471)*, PHX729 *unc-68(syb729)*, DAG862 *domEx862[unc-68p1*::*QF*::*unc-54 3’UTR;unc-68p2*::*QF*::*unc-54 3’UTR;QUAS*:: *unc-68ex13*.*1*::*mScarlet*::*unc-54 3’UTR;QUAS*::*unc-68ex13*.*0*::*BFP*::*unc-54 3’UTR]*, DAG865 *domEx865[unc-68p1*::*QF*::*unc-54 3’UTR*;*QUAS*::*unc-68ex13*::*ScarBFP*::*unc-54 3’UTR]*, DAG866 *domEx866[unc-68p1*::*QF*::*unc-54 3’UTR;unc-68p2*::*QF*::*unc-54 3’UTR;QUAS*::*unc-68ex13*::*ScarBFP*::*unc-54 3’UTR]*, DAG938 *domEx938[unc-68p1*::*QF*::*unc-54 3’UTR*;*QUAS*::*unc-68ex13*.*1*::*mScarlet*::*unc-54 3’UTR]*, DAG939 *domEx939[unc-68p1*::*QF*::*unc-54 3’UTR*;*QUAS*::*unc-68ex13*.*0*::*BFP*::*unc-54 3’UTR]*, DAG940 *domEx940[unc-68p2*::*QF*::*unc-54 3’UTR*; *QUAS*:: *unc-68ex13*.*1*::*mScarlet*::*unc-54 3’UTR]*, DAG949 *domEx949[unc-68p2*::*QF*::*unc-54 3’UTR*;*QUAS*:: *unc-68ex13*.*0*::*BFP*::*unc-54 3’UTR]*, DAG1150-1152 *domEx1150-1152[unc-68p1*::*NLS*::*mScarlet*::*unc-54 3’UTR;unc-68p2*::*mNeonGreen*::*unc-54 3’UTR]*.

S1 File presents the sequences of the genomic regions edited with CRISPR/Cas9 (Suny Biotech, Fuzhou, China). Strains PHX218 *unc-68(syb218)* and PHX220 *unc-68(syb220)* were outcrossed four times with wild type (to generate strain DAG1242 and DAG1243, respectively).

### Transcriptional reporters for *unc-68* promoter 1 and 2

The three-fragment MultiSiteGateway system (Invitrogen) was used. We first created slot 1 Entry plasmids containing either *unc-68* promoter 1 or promoter 2. To that end, we amplified N2 genomic DNA by PCR using the following primers flanked with attB4 and attB1r recombination sites:

attB4unc-68p1_F: ggggacaactttgtatagaaaagttgATCGTTGGTTAATAATTGTTGGCTAACCGT
attB1runc-68p1_R: ggggactgcttttttgtacaaacttgTCTGTAAAACAAAAAAACTAGAGGTGCTGG
attB4unc-68p2_F: ggggacaactttgtatagaaaagttgATACAAAGTTCAAGTTGACAATTAGTTCT
attB1runc-68p2_R: ggggactgcttttttgtacaaacttgTTTCTTGGAACTAACTAATCATATCACTG

PCR products were then cloned into pDONR-P4-P1R vector (Invitrogen) by BP recombination to create: dg604 *[slot1 Entry unc-68p1]* and dg603 *[slot1 Entry unc-68p2]*.

These Entry plasmids were then each recombined with a *mNeonGreen* slot2 Entry plasmid (dg398) [34], an *unc-54 3’UTR* slot3 Entry plasmid (pMH473, gift from Marc Hammarlund), and a pDEST R4R3 destination vector via LR reactions. Resulting expression plasmids were named dg575 *[unc-68p1*::*mNeonGreen*::*unc-54 3’UTR]* and dg576 *[unc-68p2*::*mNeonGreen*::*unc-54 3’UTR]*. dg735 *[unc-68prom1*::*NLS*::*mScarlet*::*unc-54UTR]* was created via a LR reaction combining dg604, dg651 *[slot2 Entry NLS*::*mScarlet]*, pMH473 and pDEST R4R3.

### Alternative splicing reporter minigenes

The different minigenes were obtained by gene synthesis (Eurofins DNA, sequences reported in S2 File) and subcloned as slot2 Entry clones compatible with the three-fragment MultiSiteGateway system (Invitrogen). The resulting Entry plasmids were:

dg642 *[slot2 Entry unc-68ex13*.*1*::*mScarlet]*
dg643 *[slot2 Entry unc-68ex13*.*0*::*CeBFP]*
dg644 *[slot2 Entry unc-68ex13*::*ScarBFP]*

The *mScarlet* sequence in dg642 was derived from the previously described codon-optimized *wrmScarlet* transgene [35], and included two artificial introns [36]. The *mScarlet* sequence in dg644, was further modified to remove stop codons that could occur in case of frame shift. The CeBFP coding sequence was that of mTagBFP[37], which had been codon-optimized. The version in dg643 included three artificial introns, while that in dg644 did not include any intron.

Each minigene was recombined downstream of a QUAS promoter via a LR reaction with dg229 *[slot1 Entry QUASprom]* [38], pMH473 *[slot3 Entry unc-54 3’UTR]* and pDEST R4R3 destination vector. Resulting expression plasmids were named:

dg645 *[QUAS*:: *unc-68ex13*.*1*::*mScarlet*::*unc-54 3’UTR]*
dg646 *[QUAS*:: *unc-68ex13*.*0*::*BFP*::*unc-54 3’UTR]*
dg647 *[QUAS*:: *unc-68ex13*::*ScarBFP*::*unc-54 3’UTR]*.

The QUAS promoter was used in the two-component Q-system, where it can be activated by the QF transcription factor, expressed under the control of a second promoter from a separate construct [39]. To generate plasmids driving the expression of QF under the control of *unc-*68 promoter 1 or promoter 2, we performed LR reactions to recombine either dg604 *[slot1 Entry unc-68p1]* or dg603 *[slot1 Entry unc-68p2]* with a *QF* slot2 Entry plasmid (dg240), an *unc-54 3’UTR* slot3 Entry plasmid (pMH473) and a pDEST R4R3 destination vector. Resulting expression plasmids were named dg648 *[unc-68p1*::*QF*::*unc-54 3’UTR]* and dg649*[unc-68p2*::*QF*::*unc-54 3’UTR]*.

When generating transgenic lines, we combined each QUAS-minigene construct with either *unc-68p1*::*QF* construct, *unc-68p2*::*QF* construct, or both of them at the same time. Our initial goal was to express these reporters in all UNC-68-expressing tissues using both promoter 1 and 2 to drive the transgene expression. However, despites numerous attempts, we never detected signal in body wall muscle, but only in neurons and pharyngeal muscle. Of note, we easily obtained transgenics when using only a promoter 2 construct, obtained no transgenic line when using only a promoter 1 construct, and obtained transgenic lines at a dramatically reduced frequency when using a mix of the two constructs. In the latter case, expression was absent in body wall muscles. A possible explanation is that our minigenes display some toxicity when expressed in body wall muscles, strongly counter-selecting transgenic animals with detectable expression in this tissue. The exon 13 tissue-specific analysis presented in the Result section is therefore limited to pharyngeal muscle and neurons.

### Transgenesis

DNA was prepared with a GenElute^TM^ HP Plasmid miniprep kit (Sigma) and was microinjected at a concentration of 20 ng/μl according to a standard protocol [40]. Either *[unc-122p*::*GFP]* or *[unc-122p*::*RFP]* were used as co-injection markers.

### Microscopy

For fluorescent reporter imaging, we used either a Zeiss Axio Plan 2 fluorescence microscope (40x air objective, NA = 0.95) or a Leica TCS SPE-II confocal microscope (APO 40x oil objective, NA1.15), equipped with a 488 nm wavelength diode laser and an ET525/50m emission filter. Z-stack images were acquired across whole animal thickness and maximal intensity projections are depicted.

### *unc-68* alternative exon combination analysis

For the analysis reported in Fig 3, a mixed stage N2 cDNA library was prepared as previously described [41] and used as a template for the PCR amplification of the 5’ region of exon 1.1- or exon 1.2-containing *unc-68* transcript isoforms, respectively, using the following primers (as illustrated in Fig 3A):

unc-68ex1.1F: aggcgaacaggatgatgtctctt
unc-68ex1.2F: tcacggatatctcagatgaggatca
unc-68ex15R: cgttccatcttctcaagagcgatt

After purification with a DNA-clean up kit (Zymogen), PCR products (~4.7 kb in size) were cloned into the pCR4-TOPO TA vector (Invitrogen), and resulting clones sequenced from the 3’ extremity to determine the specific exon combination in the DR2 region of each clone.

### SRT data analysis

For the analysis reported in Fig 2C, we recovered published tissue-specific transcriptomic data obtained with the SRT method by Ma and collaborators [24]. From the supplementary material available in this publication, we extracted transcript counts in muscle and neurons (old adult dataset) for the *unc-68* transcript. Based on genomic coordinates, we could differentiate between exon 1.1-containing and exon 1.2-containing transcripts. Raw data are available in S3 File.

### Pharyngeal pumping analysis

Videos of grinder movements of adult animals on food were recorded at a 160X magnification thanks to a stereomicroscope (Leica M2015FA) equipped with a camera (Leica DFC345FX), as previously described [42]. Grinder movements were scored manually over 20 s to determine pumping rate.

### Swimming analysis

First day adult animals were transferred to 24-well plates with M9 buffer and videos were recorded using a camera (DMK33UX250).

Motility of worms was assessed as the number of waves of body bending per min in M9 buffer. Body bending was scored using a computer assisted analysis in Image J as previously described [43].

### High-content behavioral and postural analysis in crawling animals

First day adult animals crawling on OP50 *E*. *coli* seeded NGM petri dishes were video-recorded and analyzed with the Tierpsy tracker [29, 30]. At least five plates were analyzed per genotype, each recording at least 10 animals for a duration of 15 minutes. We focused on a subset of parameters significantly diverging from wild type in at least one mutant. Statistical significance was determined with multiple Student *T*-tests, using Holm-Bonferroni correction for multiple comparisons (*P* < .01).

## Supporting information

S1 Fig Localization of alternative exons in the current *unc-68* gene model. (PDF)

S2 Fig *unc-68* promoter analysis with two-color reporters. (PDF)

S3 Fig Alignment of UNC-68 and human RyR protein sequences around DR2. (PDF)

S4 Fig *unc-68* DR2 genomic sequence alignment across *Caenorhabditis* species. (PDF)

S5 Fig Phenotypic comparison between non-outcrossed and outcrossed strains carrying deletions in *unc-68* exon 10 and 13. (PDF)

S1 File Description of the genome editions made in this study. (PDF)

S2 File Sequences of the *unc-68* splicing reporter minigenes. (PDF)

S3 File Raw data used for tissue-specific SRT analysis. (PDF)
