# Supplementary material for: Tissue-specific isoforms of the single C. elegans Ryanodine receptor gene unc-68 control specific functions
Source: PLoS Genet. 2020 Oct 26;16(10):e1009102. doi: 10.1371/journal.pgen.1009102 (PMC7644089; doi:10.1371/journal.pgen.1009102)
Supplement: S1 Fig — (PDF) [file pgen.1009102.s001.pdf]

S1 Figure

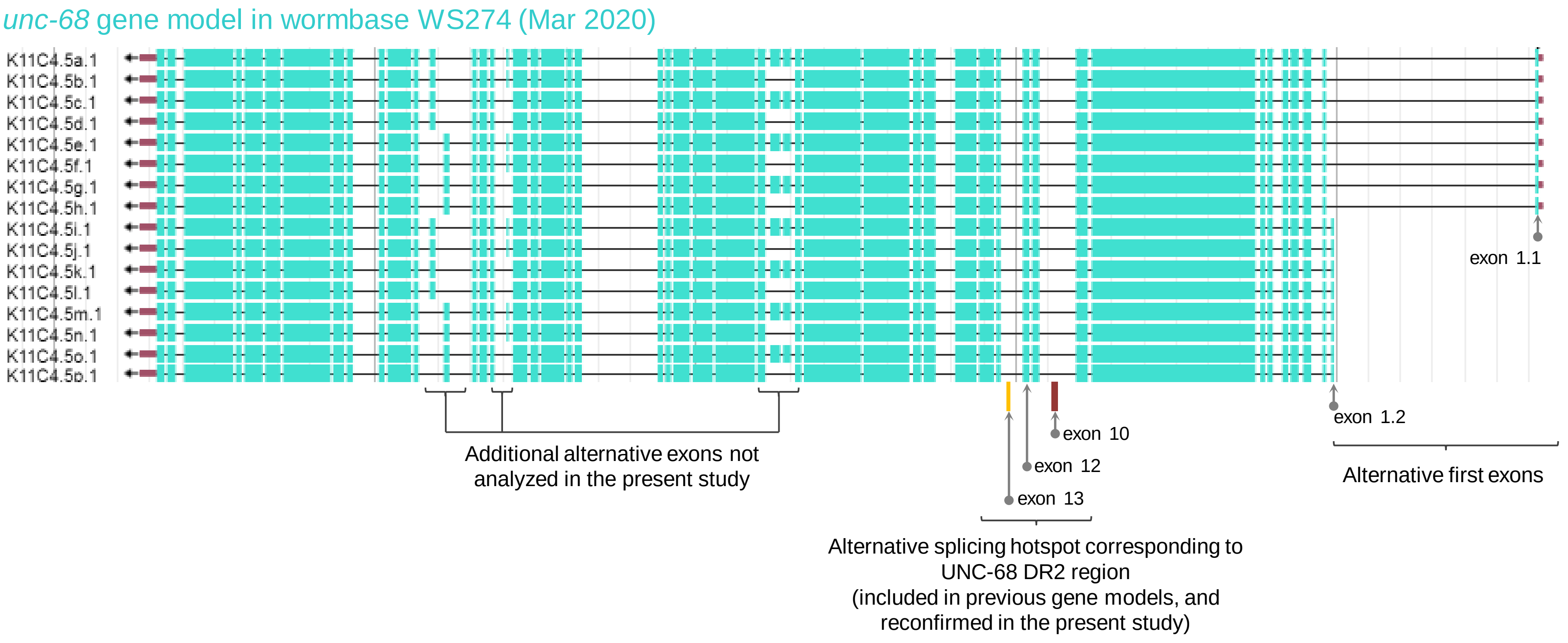

S1 Figure. Localization of alternative exons in the current *unc-68* gene model
