# Supplementary material for: Tissue-specific isoforms of the single C. elegans Ryanodine receptor gene unc-68 control specific functions
Source: PLoS Genet. 2020 Oct 26;16(10):e1009102. doi: 10.1371/journal.pgen.1009102 (PMC7644089; doi:10.1371/journal.pgen.1009102)
Supplement: S2 Fig — (PDF) [file pgen.1009102.s002.pdf]

S2 Figure

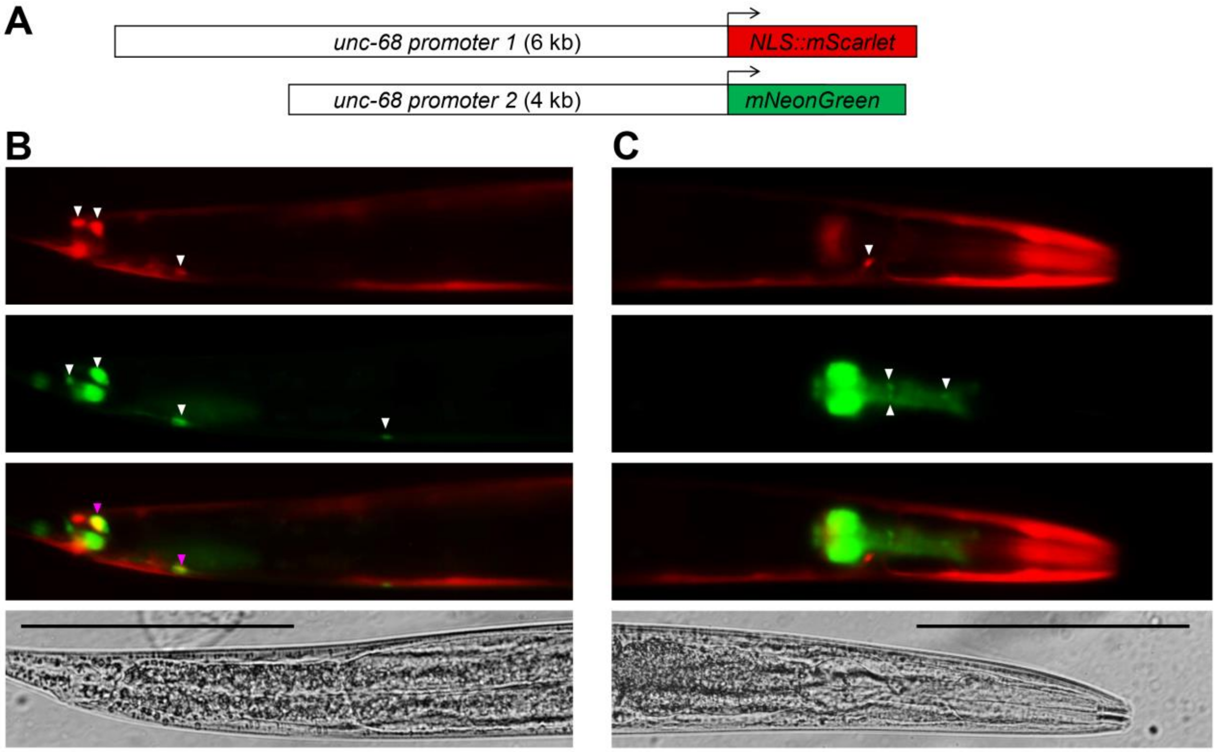

S2 Figure. *unc-68* promoter analysis with two-color reporters
(A) Schematic of the two co-injected reporter genes.
(B, C) Representative micrographs of tail (B) and head (C) regions. From top to bottom: red channel, green channel, merged red/green channel, and DIC. Similar results were obtained in three independent transgenic lines. The Nuclear Localization Signal (NLS) in the *[unc-68p1::NLS::mScarlet]* did not generate a strong nuclear accumulation of the red fluorescent protein. White arrowheads: cells expressing either reporter. Pink arrowheads: cells expressing both reporters. Scale bars: 100 µm
