# Supplementary material for: Tissue-specific isoforms of the single C. elegans Ryanodine receptor gene unc-68 control specific functions
Source: PLoS Genet. 2020 Oct 26;16(10):e1009102. doi: 10.1371/journal.pgen.1009102 (PMC7644089; doi:10.1371/journal.pgen.1009102)
Supplement: S3 Fig — (PDF) [file pgen.1009102.s003.pdf]

S3 Figure

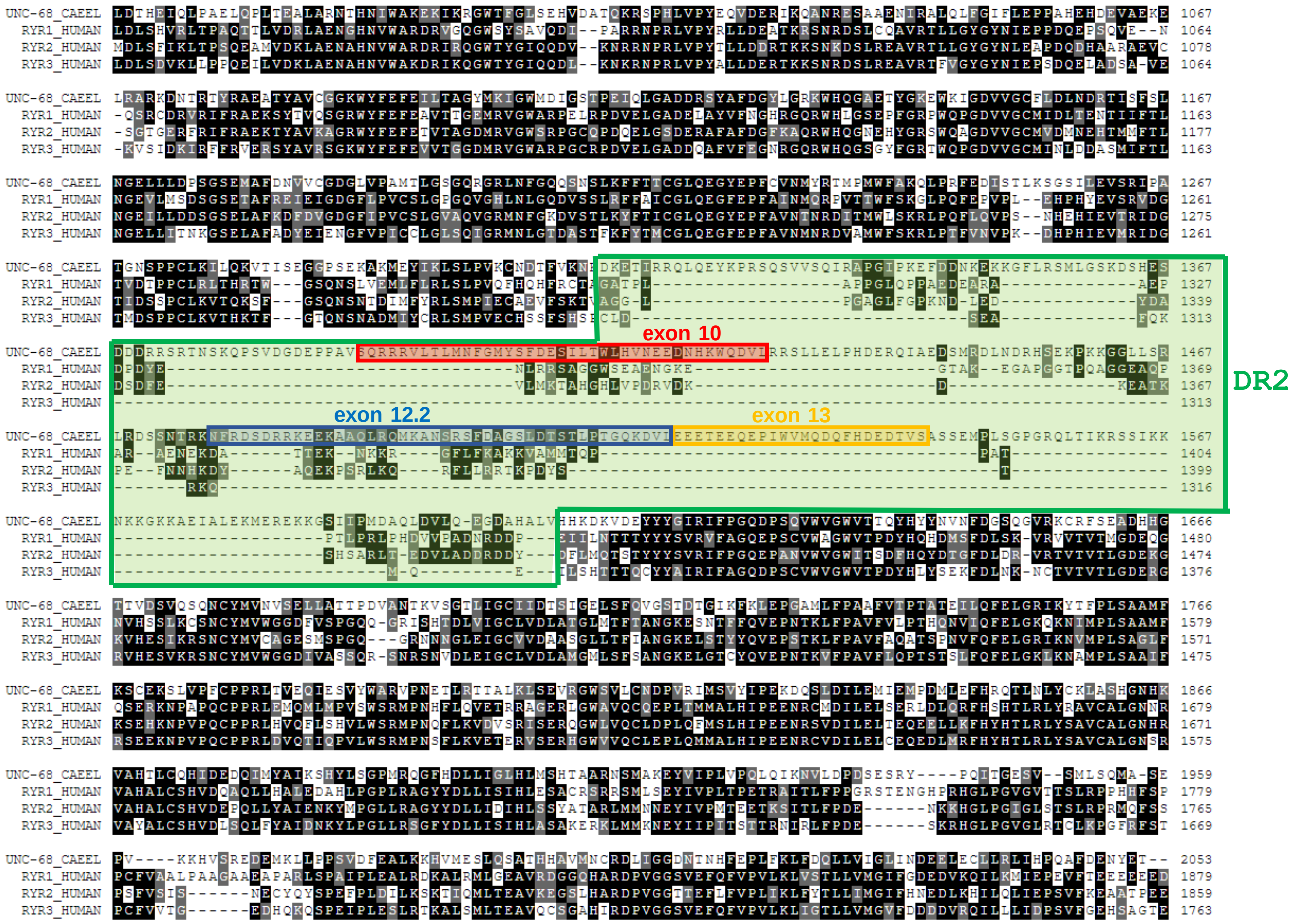

S3 Figure. Alignment of UNC-68 and human RyR protein sequences around DR2

Alignment of the *C. elegans* UNC-68 protein sequence (theoretical isoform containing exon 10, 12.2 and 13), with human RyR1, RyR2, and RyR3 sequences. Only the part around DR2 is depicted. The DR2 region is highlighted in green, and the sequence stretches encoded by exons 10, 12.2 and 13 in *unc-68* are highlighted in red, blue and yellow, respectively.
