# Supplementary material for: Tissue-specific isoforms of the single C. elegans Ryanodine receptor gene unc-68 control specific functions
Source: PLoS Genet. 2020 Oct 26;16(10):e1009102. doi: 10.1371/journal.pgen.1009102 (PMC7644089; doi:10.1371/journal.pgen.1009102)
Supplement: S4 Fig — (PDF) [file pgen.1009102.s004.pdf]

S4 Figure

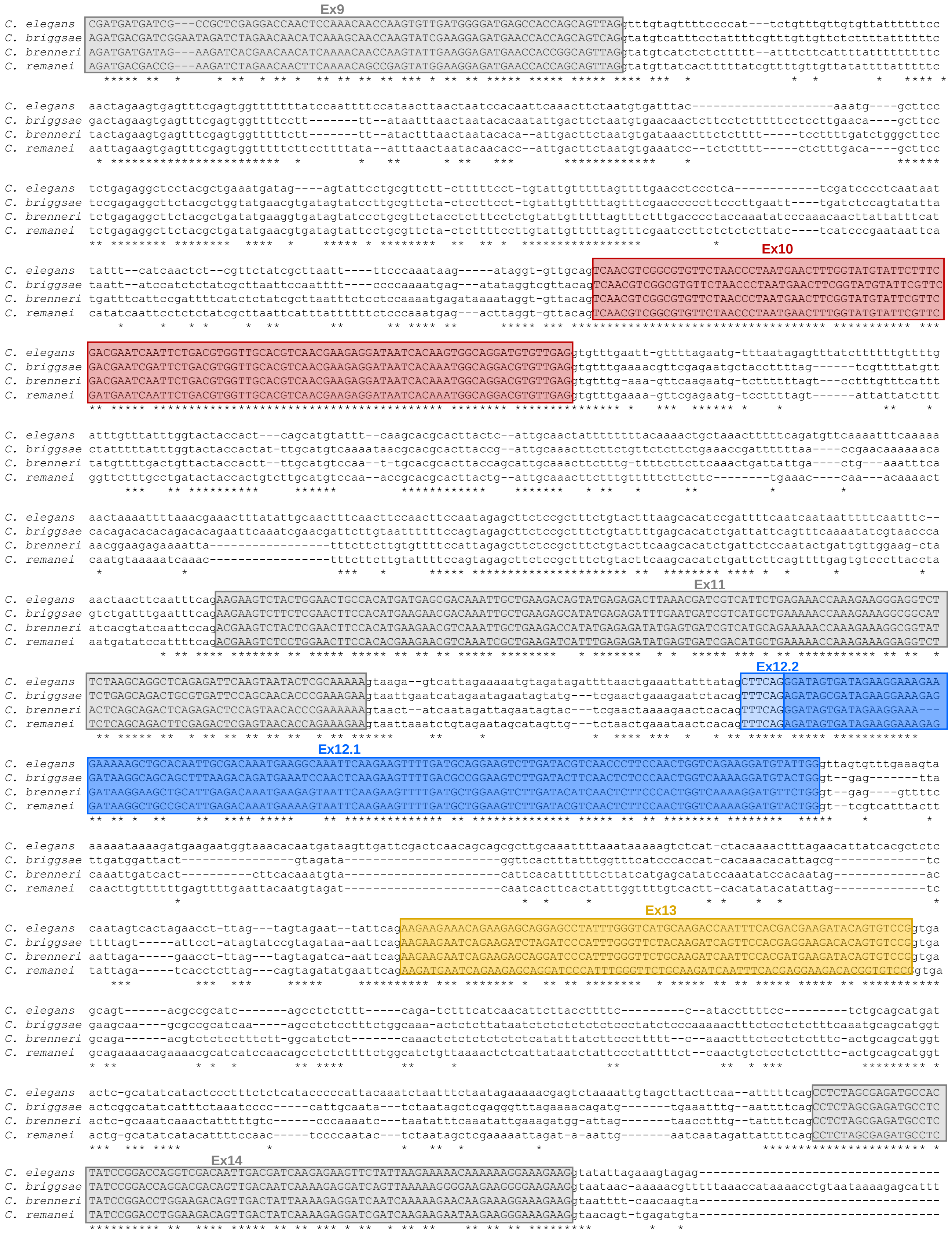

Figure S4: *unc-68* DR2 genomic sequence alignment across *Caenorhabditis* species

Alignment of the *C. elegans unc-68* genomic sequence with the homologous sequences from the indicated *Caenorhabditis* species. Only the region coding for the variable part of DR2 and nearby sequence is depicted. Alternate exons 10, 12 and 13 are highlighted in red, blue, and yellow, respectively. Two shades of blue are used to highlight the short exon 12.1 (dark blue) and the extra 6 nucleotides retained only in the long exon 12.2 (light blue). Constitutive exons 9, 11, and 14 are highlighted in grey.
