# Supplementary material for: Tissue-specific isoforms of the single C. elegans Ryanodine receptor gene unc-68 control specific functions
Source: PLoS Genet. 2020 Oct 26;16(10):e1009102. doi: 10.1371/journal.pgen.1009102 (PMC7644089; doi:10.1371/journal.pgen.1009102)
Supplement: S5 Fig — (PDF) [file pgen.1009102.s005.pdf]

# S5 Figure

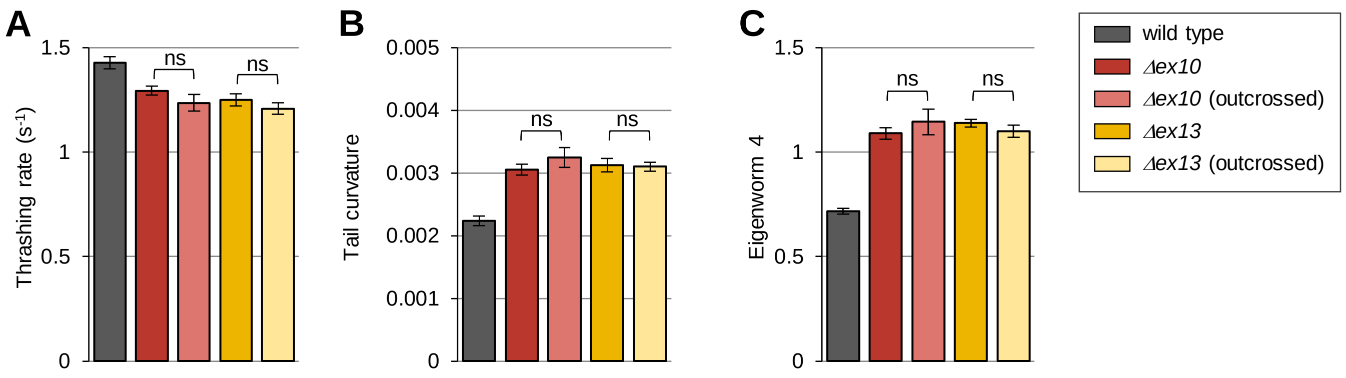


**S5 Figure. Phenotypic comparison between non-outcrossed and outcrossed strains carrying deletions in *unc-68* exon 10 and 13**

Phenotypic analyses of wild type (N2) and the indicated engineered mutants (see mutation description in Fig. 6A). The comparison was made between non-outcrossed lines and lines that had been outcrossed four times with wild type (outcrossed). Results as means (+/- s.e.m). $n \geq 47$ animals (A); $n=7$ plates (each with at least 10 tracked animals (B-C)). ns, not significant by Student's *t*-tests.
