# Supplementary material for: Tissue-specific isoforms of the single C. elegans Ryanodine receptor gene unc-68 control specific functions
Source: PLoS Genet. 2020 Oct 26;16(10):e1009102. doi: 10.1371/journal.pgen.1009102 (PMC7644089; doi:10.1371/journal.pgen.1009102)
Supplement: S1 File — (PDF) [file pgen.1009102.s006.pdf]

S1 File. Description of the genome editions made in this study

Strain: PHX214

Allele: *syb214*

Description: deletion of *unc-68* promoter1 and exon 1.1

Wild type sequence (with deletion in double strikethrough):

ttgatagaaggatataccatataaaatttcatatgtttcagaaaacttcgagatttcaatcacaacgattttctatggggaatgagagcaaga
aaggaaatttatgacaaaataataaacaccatcaaattggatcaaagaagagtgaagttgcaaaatagtatggagagattctttgaaagt
gagtttcagatattttgatgtcgttttgaaaattgttaacagaatggaatcagaacatcttcgttttatattggaaatttttctgtaaactaaaacc
tcaaaaaaacaaattatgttaaaattttattctcaaaacactagtagttgctataaagttgaagtttgaaggaatttgacataaaatgctcaaa
acttccaataatttttttgataggttaaatttagattgaaaaattaaggtgttgcctatttccgaattaatgaatttcgatttgaagttttttactggc
ccaactttttccaaattccaaatattattttcagggcaatcacgaaattcaacttccggtcttgatgaagaaacgatgatgaggttacgaaat
gaaacgatgaatctagactaaaacgaatttgttgttttgatttctttttttttaaagtagttttcagatctcttccagatactatttcgtaatagttgt
ttcc~~cttcacatatgaaactgatcgggtttttattgtttttatcatgaataaatcactcaaaacattagttgtttgtgttttccacgtctgcgccttg
ctcctggccaccgcgagcaggacgccgctctttttcacttgagcctttcggcgcgactaataaatgatcccgcggacgcgagagcccc
caagcgcaaagtatccatccgcgatcgagcacaaagatatacacttcacacactctccacatctcggacagagataatgatttttgtgtttt
atagtttttttacttcttaatttctctattaataacattaattcttccatattgtagaaaatatgtagatcaaaataccaaatcatcaaaagttttattg
aattgccaaattgaaactgtaatttttgagtaaagctgacaattttaacgctaacaaggtatatatacaaatatattgaatttctcacaatttaca
atcaacgttttggtaaattcccaaaattacaaaaattggaattttgcgaaatttagagcattgacgcttgttttcacctgtatttgtcatttttttga
ccgtaaatcaaaactttcaaacatttttgaaacgtgtgactgtagtattcaactatttcagccactttcaaagcatctatcaatttggtaaaaac
gtttaattttaactgtagagaagctcactaatccgtccaacttgtaatttatgcttatttgcaaaaactgctttatttgcatgaattccttctttact
aaatttttcaaatttttcctaaacccacagcaactgaacccacagcaaccggtgttccaacaaaaaatgagtcaacgtcactggtgcaca
tctgcattcataataatcttcttcttttacttctcatccaccccactcggaaacttcatctttctatctctctctttccctattccatcacctgcaaga
gagcacgagacgcagccgtatcgtcggcaatggctattttacactgtattccgatgaaagacaattactttagcgaggtatcagataga
cagagacagaactcgaaccgcggctgttaagacgaagagctggcgggctcctgccaccgagaagtcgaggagagagaagatccgt
tggcaggcaggggcagctgtattcttctacttttcatcttcttgttctagttgacgttttcttaggtgtgtgtgacgtggcgtaaggtcacataa
ttgcctagttcttgcacaagacaaaactgaaacgtctacttgtgtgtcccataaaaattgttagaaaaaaaaagataatttccaactattattt
aaaactgcagtagtttcaaatcttacgtaaaaactacatacataacttacacgttgaaattaccgaacaatttttattttacagaagccacgttt
tgtctgaaacaattgaaaatacatagtaaaactgacattgagaactataatataatattgagaactatattatgagaactatgatgaaactttc
aaaaatgtttatttttttggagttctcaatcaaagttggataaacacgacttaagtttttagatattttaggcgcaaatcgcagctatgatataagt
tttattatttttcatttacaaaatcgtttttctcactgcatcttccaaaatggctcaagaaaacaatagaaacccacaatctttttttcgcatatcatt
aatccgacgcagatttttacactctttcatctttgctgtttttttttccatcgtctaagatcttgtcatcgtcaatgaatatcaaattcatttagtatgt
atatacatatttctatggtttactcatccttactttacaaataataatcaaatttgtattctcttttctcgtccttcatctctccgccgtcattcaccta
tttctctcttatcctgagattagacttgagcataacaccgcagcaataaaaccaccggagagtttaattagaaaccacaattcctcctctttttt
ctcggttttgtgcttcttcctccactcatgttttacgttttatttgttactttcgacatgacttttattctgaatttcgggaggaagtatttaagaaaa
attgatgctttacacggactatttgttaaaagcgctaacatgcaaatgttggatttaatgaagtaagaaagctatactataaatcccaaatctc
agctcagtgaatatgctttaaaataaaaattaactcaaatcagagcagtttttgaatttttcaactctcttcaaaaatattaaacgcacgttaa
attttgtattgaaggaaatattattcactttactgcttgcacttgaaaatagtatttctacttttccactttaaaaaatctcaaaaacctccttggca
ataaatctaaaatatccccataattttccgttcgtttttctgtcgtcttttgctcccgtttgttattttttttttgcgttttcccaagtggtaccagca
cctctagtttttttgttttacagatgcaaaaccagtgaaaaacggaacgaactgagtgagcagatataagaacgaaaccgaccgaacatt
atcagatcgacgatcgacaacgatggccgacaaggaggagcagggaggaggcgaacaggatgatgtctcttttctccgaacggtt~~ag
ttttattcaaaacagacaaagttcaagttgacaattagttctccggttgacttttcggaggggaatatttcggctctcacttgaatgtagatcg
ataacatcgataacattttgaaatattaatttctgaattttttttttgcatttaattgtgtagtacggaatgttgtacttttcaaattgtgcaaaaatgt
gccacctcgcttaaacgagtgaaaagaagaggaccctccggggtgtaagtgcgcgattcgcaaatctcgtaaaaagagaaacgacaa
aagagaaaaagcactctccgcatatccctgtttgattgtaacataaagtcgttttgtttccgtatagtttaaaatttttttgaatactctcctacctt
atgaaatgaattcaattctactacaattgagcatgtgtgaaagtttatagtaaccgaaaaacttaccattgtatttttgctcaagtcaaaatcta
gtaagatttctagtaaaagctcccatcttaatgataagctttcaaaaatctgctttcactcggtcggaggcgcctctcacccgcctcgcttcc

cagtgagcaaactgtcgttacagtgtttgcgggataaaaatacatataaagaacccaagtatgaaacacgtaattcaatgttgtcattacgt
tgcagcgtgaaaagaaaatatggtacttatgtattacatgccctcattttattagtaattttttaagccatgaaaaataaaaacgtgaaccaca
atttgtatttttagacttagg

Edited sequence (left and right of the deletion with a different color):

ttgatagaaggatataccatataaaatttcatatgtttcagaaaacttcgagatttcaatcacaacgattttctatggggaatgagagcaaga
aaggaaatttatgacaaaataataaacaccatcaaattggatcaaagaagagtgaagttgcaaaatagtatggagagattctttgaaagt
gagtttcagatattttgatgtcgttttgaaaattgttaacagaatggaatcagaacatcttcgttttatattggaaatttttctgtaaactaaaacc
tcaaaaaaacaaattatgttaaaattttattctcaaaacactagtagttgctataaagttgaagtttgaaggaatttgacataaaatgctcaaa
acttccaataattttttgataggttaaatttagattgaaaaattaaggtgttgcctatttccgaattaatgaatttcgatttgaagtttttttactggc
ccaactttttccaaattccaaatattattttcagggcaatcacgaaattcaacttccggtcttgatgaagaaacgatgatgaggttacgaaat
gaaacgatgaatctagactaaaacgaatttgttgttttgatttcttttttttttaaagtagttttcagatctcttccagatactatttcgtaatagttgt
ttccagttttattcaaaacagacaaagttcaagttgacaattagttctccggttgacttttcggaggggaatatttcggctctcacttgaatgta
gatcgataacatcgataacattttgaaatattaatttctgaattttttttttgcatttaattgtgtagtacggaatgttgtacttttcaaattgtgcaaa
aatgtgccacctcgcttaaacgagtgaaaagaagaggaccctccggggtgtaagtgcgcgattcgcaaatctcgtaaaaagagaaac
gacaaaagagaaaaagcactctccgcatatccctgtttgattgtaacataaagtcgttttgtttccgtatagtttaaaattttttgaatactctcc
taccttatgaaatgaattcaattctactacaattgagcatgtgtgaaagtttatagtaaccgaaaaacttaccattgtatttttgctcaagtcaa
aatctagtaagatttctagtaaaagctcccatcttaatgataagctttcaaaaatctgctttcactcggtcggaggcgcctctcacccgcctc
gcttcccagtgagcaaactgtcgttacagtgtttgcgggataaaaatacatataaagaacccaagtatgaaacacgtaattcaatgttgtc
attacgttgcagcgtgaaaagaaaatatggtacttatgtattacatgccctcattttattagtaattttttaagccatgaaaaataaaaacgtga
accacaatttgtatttttagacttagg

Strain: PHX215

Allele: *syb215*

Description: Deletion of *unc-68* exon 1.1

Wild type sequence (with deletion in double strikethrough):

tttctcgtccttcatctctccgccgtcattcacctatttctctcttatcctgagattagacttgagcataacaccgcagcaataaaaccaccgg
agagtttaattagaaaccacaattcctcctctttttctcggttttgtgcttcttcctccactcatgttttacgttttatttgttactttcgacatgacttt
tattctgaatttcgggaggaagtatttaagaaaaattgatgctttacacggactatttgttaaaagcgctaacatgcaaatgttggatttaatg
aagtaagaaagctatactataaatcccaaatctcagctcagtgaatatgctttaaaataaaaattaactcaaatcagagcagtttttgaatttt
ttcaactctcttcaaaaatattaaacgcacgttaaattttgtattgaaggaaatattattcactttactgcttgcacttgaaaatagtatttctactt
ttccactttaaaaaatctcaaaaacctccttggcaataaatctaaaatatccccataattttccgttcgtttttctgtcgtcttttgctcccgtttg
ttattttttttttgcgttttcccaagtggtaccagcacctctagttttttgttttacag~~atgcaaaaccagtgaaaaacggaacgaactgagtg~~
~~agcagatataagaacgaaaccgaccgaacattatcagatcgacgatcgacaacgatggccgacaaggaggagcagggaggagc~~
~~gaacaggatgatgtctcttttctccgaacggtt~~agttttattcaaaacagacaaagttcaagttgacaattagttctccggttgacttttcgga
ggggaatatttcggctctcacttgaatgtagatcgataacatcgataacattttgaaatattaatttctgaattttttttgcatttaattgtgtagt
acggaatgttgtacttttcaaattgtgcaaaaatgtgccacctcgcttaaacgagtgaaaagaagaggaccctccggggtgtaagtgcg
cgattcgcaaatctcgtaaaaagagaaacgacaaaagagaaaaagcactctccgcatatccctgtttgattgtaacataaagtcgttttgtt
tccgtatagtttaaaattttttgaatactctcctaccttatgaaatgaattcaattctactacaattgagcatgtgtgaaagtttatagtaaccga
aaaacttaccattgtatttttgctcaagtcaaaatctagtaagatttctagtaaaagctcccatcttaatgataagctttcaaaaatctgctttca
ctcggtcggaggcgcctctcacccgcctcgcttcccagtgagcaaactgtcgttacagtgtttgcgggataaaaatacatataaagaac
ccaagtatgaaacacgtaattcaatgttgtc

Edited sequence (left and right of the deletion with a different color):

tttctcgtccttcatctctccgccgtcattcacctatttctctcttatcctgagattagacttgagcataacaccgcagcaataaaaccaccgg
agagtttaattagaaaccacaattcctcctctttttctcggttttgtgcttcttcctccactcatgttttacgttttatttgttactttcgacatgacttt
tattctgaatttcgggaggaagtatttaagaaaaattgatgctttacacggactatttgttaaaagcgctaacatgcaaatgttggatttaatg
aagtaagaaagctatactataaatcccaaatctcagctcagtgaatatgctttaaaataaaaattaactcaaatcagagcagtttttgaatttt
ttcaactctcttcaaaaatattaaacgcacgttaaattttgtattgaaggaaatattattcactttactgcttgcacttgaaaatagtatttctactt
ttccactttaaaaaatctcaaaaacctccttggcaataaatctaaaatatccccataattttccgttcgtttttctgtcgtcttttgctcccgtttg
ttattttttttttgcgttttcccaagtggtaccagcacctctagttttttgttttacagagttttattcaaaacagacaaagttcaagttgacaatt
agttctccggttgacttttcggaggggaatatttcggctctcacttgaatgtagatcgataacatcgataacattttgaaatattaatttctgaa
ttttttttgcatttaattgtgtagtacggaatgttgtacttttcaaattgtgcaaaaatgtgccacctcgcttaaacgagtgaaaagaagagga
ccctccggggtgtaagtgcgcgattcgcaaatctcgtaaaaagagaaacgacaaaagagaaaaagcactctccgcatatccctgtttg
attgtaacataaagtcgttttgtttccgtatagtttaaaattttttgaatactctcctaccttatgaaatgaattcaattctactacaattgagcatg
tgtgaaagtttatagtaaccgaaaaacttaccattgtatttttgctcaagtcaaaatctagtaagatttctagtaaaagctcccatcttaatgat
aagctttcaaaaatctgctttcactcggtcggaggcgcctctcacccgcctcgcttcccagtgagcaaactgtcgttacagtgtttgcggg
ataaaaatacatataaagaacccaagtatgaaacacgtaattcaatgttgtc

Strain: PHX216

Allele: *syb216*

Description: deletion of *unc-68* promoter2 and exon 1.2

Wild type sequence (with deletion in double strikethrough):

actataaatcccaaatctcagctcagtgaatatgctttaaaataaaaattaactcaaatcagagcagtttttgaatttttcaactctcttcaaaa
atattaaacgcacgttaaattttgtattgaaggaaatattattcactttactgcttgcacttgaaaatagtatttctacttttccactttaaaaaatc
tcaaaaacctccttggcaataaatctaaaatatccccataattttccgttcgtttttctgtcgtcttttgctcccgtttgttatttttttttgcgtttt
cccaagtggtaccagcacctctagttttttgttttacagatgcaaaaccagtgaaaaacggaacgaactgagtgagcagatataagaac
gaaaccgaccgaacattatcagatcgacgatcgacaacgatggccgacaaggaggagcagggaggaggcgaacaggatgatgtct
cttttctccgaacg~~gttagttttattcaaaacagacaaagttcaagttgacaattagttctccggttgacttttcggaggggaatatttcggct~~
~~ctcacttgaatgtagatcgataacatcgataacattttgaaatattaatttctgaatttttttttgcatttaattgtgtagtacggaatgttgtactttt~~
~~caaattgtgcaaaaatgtgccacctcgcttaaacgagtgaaaagaagaggaccctccggggtgtaagtgcgcgattcgcaaatctcgt~~
~~aaaaagagaaacgacaaaagagaaaaagcactctccgcatatccctgtttgattgtaacataaagtcgttttgtttccgtatagtttaaaatt~~
~~ttttgaatactctcctaccttatgaaatgaattcaattctactacaattgagcatgtgtgaaagtttatagtaaccgaaaaacttaccattgtattt~~
~~ttgctcaagtcaaaatctagtaagatttctagtaaaagctcccatcttaatgataagctttcaaaaatctgctttcactcggtcggaggcgcc~~
~~tctcacccgcctcgcttcccagtgagcaaactgtcgttacagtgtttgcgggataaaaatacatataaagaacccaagtatgaaacacgt~~
~~aattcaatgttgtcattacgttgcagcgtgaaaagaaaatatggtacttatgtattacatgccctcattttattagtaatttttaagccatgaaa~~
~~aataaaaacgtgaaccacaatttgtatttttagacttaggaaataacattttctaagcctaaagaggaaaaatgtatatcacgtttattttaact~~
~~agagaaattaatgacaatttaatttaatttgtaaatgagggcatgtaatacacaagtaccgaaaatattggaagtgggttcaattcacgtgtt~~
~~ctatattttcatttctcgttaaaaacaacgtttatttttcggaggtttttgtatggcgtagaagatggtcaggctgtttttcaaacattggttagtt~~
~~gtggtcaagtatatttgttacaatgtttgatttatatttattaaatttccagaggtatagttagatatctcaaaattcttatcgttggattttacaatta~~
~~agatattaatttattccgcgacttcttctctgtttattcatttactcgtatgcaaaaaaactgaactttaaccttttttgaatacgcgtgcgaattga~~
~~gatgctaagaaaggaagtatgagagtgagagagaaggattgaatccaaaaattccgaaatgttttgggtgggagccggacccccaatg~~
~~aatgtaatccggtgacgagaacgctgccgatttggcacatttaatcaacaatttatagccggatggcgttgagtgtgaaatagagggtga~~
~~gagaaaaagagtgagatgaagtgacgaagaaaacgagatttttggcagagtcagaggctcttctgacaaactacctaccctctcttggc~~
~~aaccactactttttgcccatgtttgcttgaactcgaaacaaaaaaatttagggggatatttgcgtgttttatgtgcttccgcatacattcggatca~~
~~tctcgaatttctttcctgccaacacttgttttgaatttttggggttttagtaaaagtttttttccgcaattcttagtcagagtagatagttcttgcatt~~
~~aatgttttggtaacatctgccagctaaaaatcatccaaaatttgtaaagttgatacttgtcgaagattacacgacgactctctgttagtcaaac~~
~~ttctataattttcaaaagcataagaattttgattgcaattttctagtttctcaaaatttgagacatttctacatagcatgcacacttcaactctaaat~~
~~taagaacaatatgatgtctgactgaaaatttgtgtcttcaccctactagatacaattttgcaacaaaatatgctgcacgcatgttgaaattga~~
~~gagaaacacttcttgtcattcgcttttttttggttttatgacaacgcagatgaaatagtaaaatgacgaagaaaaaaaggaagaaaataatt~~
~~gaaatttgtgcatatatgagtggtgcccagggtggcgctagtctcactctcaccctcccgtccttcccctacacacatcttttcttaaatgtat~~
~~gaaaagagagaacttgatggggcaaaagcatgcgattctgagagacttagagagctgacacttttgctcgtagacgtcgtgtttcacttc~~
~~gtttcgctccccaccctgcctcctacttcttgatttgtttcggctttttctagtcaaattagtattaggtgacattgaccttgtagctagccgcctc~~
~~tctgagcttagaaaccccagagattagtttttaactattgctctgcttgttatttaccaatcaaaaaatgtttttgaaacaaaaaagctctgtcg~~
~~aagcatcaaaaaagcaaaaaaatacgcggttaacttaaaaaaaggcgagcacccggcgtcggatcctattcagttcctttccgtctcttc~~
~~tcttcttccccctttggcctcgttttgtgcattcatctcccgtccgccttccaactttttgtcctctcctacgcacatacaacccataaacgtatttc~~
~~tgggacacagctatttttgaatagaccctttccggattagcctttttgaagcctgtgattattgttttcattaaatgcttaacttatcaagtgcaa~~
~~cacttctttttgattttttaaacataaacctgcagaacacaaactttgaaaatttcgaaaaactcattttgttgctttaaattttgtgtgggccgta~~
~~catatttaataggagtcacactaattcatttttaataggacacaatgcaattgtttcgaatttttgacaaattaccctctcatattgtcacgcaac~~
~~atgtgaatatgccagcgttttctttttttttcgaaacagacccagcaagggatcaattacctagaattatcaaaagttgaagggaggcgggt~~
~~gactcttttgatggatttacgaaattttgattcggacaggtatatgcgacaggaatctgagcaaatcgaatctgcaagagagtgcagtgta~~
~~ctatgagagcgcagcttgatagtgatctcacttagaaaacaaacaagctgctcctggcttttgccgatttgatctcgcccaccaaggcttc~~
~~atcttcccatttccatcgttttccattattttgcacaacaaaacttgaaatttttcaaaaaggggctctgtttgcaaaaagcgagaaaactgaa~~
~~ctcaaaaattcaaactcctagagcaagtggtgaattttcagataactttttattttcccatattaattcagtttccacgtgttgaaaaaatttcga~~
~~atttcgtgcagaaaattgcaaaaaaagttgtttgtttttcaaaatttaaataaaagttgcatgttttcttagaaatccttgatccaaatttaagtct~~
~~aacgtctttcttcaatcaataaaaaaatatctagatatagattttctcgatttttctctgagatcccttcaaaactacaactccagtctactccttc~~

~~cgtgctattactacgataatcctaaaatattacaaatcatcccatataagtataagtcctctagtttcctccttctcccgatctctcttcgttttcc~~
~~ctcgagagacttatgtcgcccatatggcattcagccgtcccccggtcccgcctattgtttttcgtcgctctttttttggctcacaatttttggt~~
~~gactcttcagttttcagacaaagagactccacattagaagtctgctctaggcagcaaactcatagtattttgccaaacaagcatccgcaga~~
~~caaatacacgcaaacacaattacaacgcagatataaatatattattcagtgatatgattagttagttccaagaaatggattttgatttcacgg~~
~~atatctcagatgaggatcataactatgttacaaaggtttgtttaaaagaactattctatattaaacggaaagtctggaaatcacagaggaca~~
~~gaggaaatgaatttctttatttttgataaaatttttatttccag~~ggagacatcgtctgcctctcctgcgttgcctcacacaatagagatggagtt
ctcgggtcagaaaggttggtgacatttttgggaaactttttcttgagaagttgggcctaatataattattttttaaaaagttttttgagccgaaa
atgttttcagaagacgatctattgagaaattaaagttgtggatatacggtatccaaaaacatcagaatcagcgcagttcttgatactggagc
tcaagaacaacaatttaaaaaattataagtttttccacaataatttcagagtttgtttgtgcaccgaaggctttggaaaccggatgtgcacac
tggaaaacgtgtcggacaaggacatcccgccagatattgcaatgtgcatgctttacatcgataacgcactgtcgatgagagcattacag
gaaatgatgtcagcggatagtgatcatgtgagttttttttaattaaaaaataatttttcgcatataatggaaagatacagtt

Edited sequence (left and right of the deletion with a different color and synonymous mutations are labelled in purple):

actataaatcccaaatctcagctcagtgaatatgctttaaaataaaaattaactcaaatcagagcagtttttgaatttttcaactctcttcaaaa
atattaaacgcacgttaaattttgtattgaaggaaatattattcactttactgcttgcacttgaaaatagtatttctacttttccactttaaaaaatc
tcaaaaacctccttggcaataaatctaaaatatccccataattttccgttcgtttttctgtcgtcttttgctcccgtttgttattttttttttgcgtttt
cccaagtggtaccagcacctctagtttttttgttttacagatgcaaaaccagtgaaaaacggaacgaactgagtgagcagatataagaac
gaaaccgaccgaacattatcagatcgacgatcgacaacgatggccgacaaggaggagcagggaggaggcgaacaggatgatgtct
ctttt**cta**cga**acc**ggagacatcgtctgcctc**tcg**tgcgttgcctcacacaatagagatggagttctcgggtcagaaaggttggtgacat
tttgggaaactttttcttgagaagttgggcctaatataattattttttaaaaagttttttgagccgaaaatgttttcagaagacgatctattgaga
aattaaagttgtggatatacggtatccaaaaacatcagaatcagcgcagttcttgatactggagctcaagaacaacaatttaaaaaattata
agtttttccacaataatttcagagtttgtttgtgcaccgaaggctttggaaaccggatgtgcacactggaaaacgtgtcggacaaggacat
cccgccagatattgcaatgtgcatgctttacatcgataacgcactgtcgatgagagcattacaggaaatgatgtcagcggatagtgatca
tgtgagttttttttaattaaaaaataatttttcgcatataatggaaagatacagtt

Strain: PHX217

Allele: *syb217*

Description: Deletion of *unc-68* exon 1.2

Wild type sequence (with deletion in double strikethrough):

aaactcatagtattttgccaaacaagcatccgcagacaaatacacgcaaacacaattacaacgcagatataaatatattattcagtgatatg
attagttagttccaagaa~~atggattttgatttcacggatatctcagatgaggatcataactatgttacaaaggtttg~~tttaaaagaactattctat
attaaacggaaagtctggaaatcacagaggacagaggaaatgaatttctttatttttgataaaatttttatttccag

Edited sequence (left and right of the deletion with a different color):

aaactcatagtattttgccaaacaagcatccgcagacaaatacacgcaaacacaattacaacgcagatataaatatattattcagtgatatg
attagttagttccaagaatttaaaagaactattctatattaaacggaaagtctggaaatcacagaggacagaggaaatgaatttctttattttt
gataaaatttttatttccag

Strain: PHX218

Allele: *syb218*

Description: Deletion of alternative *unc-68* exon 10

Wild type sequence (with deletion in double strikethrough):

actccaaacaaccaagtgttgatggggatgagccaccagcagtt~~aggtttgtagttttccccattctgtttgttgtgttattttttccaactagaagtgagtttcgagtggtttttttatccaattttccataacttaactaatccacaattcaaacttctaatgtgatttacaaatggcttcctctgagaggctcctacgctgaaatgatagagtattcctgcgttcttctttttccttgtattgtttttagttttgaacctcccctcatcgatcccctcaataattatttcatcaactctcgttctatcgcttaattttcccaaataagataggtgttgcagtcaacgtcggcgtgttctaaccctaatgaactttggtatgtattctttcgacgaatcaattctgacgtggttgcacgtcaacgaagaggataatcacaagtggcaggatgtgttgaggtgtttgaattgttttagaatgtttaatagagtttatctttttgttttgatttgtttatttggtactaccactcagcatgtatttcaagcacgcacttactcattgcaactattttttttacaaaactgctaaactttttcagatgttcaaaatttcaaaaaaactaaaattttaaacgaaactttatattgcaactttcaacttccaacttccaatagagcttctccgctttctgtactttaagcacatccgattttcaatcaataatttttcaatttcaactaacttcaatttc~~agaagaagtctactggaactgccacatgatgagcgacaaattgctgaagacagtatgagagacttaa

Edited sequence (left and right of the deletion with a different color and synonymous mutations are labelled in purple):

actccaaacaaccaagtgttgatggggatgagcca**ccagcg**gttagaagaagtcta**ctc**gaactg**cca**ca**c**gatgagcgacaaattgctgaagacagtatgagagacttaa

Strain: PHX219

Allele: *syb219*

Description: Deletion of alternative *unc-68* exon 12.2

Wild type sequence (with deletion in double strikethrough):

tagatagattttaactgaaattatttatag~~cttcag~~ggatagtgatagaaggaaagaagaaaaagctgcacaa

Edited sequence (left and right of the deletion with a different color and synonymous mutations are labelled in purple):

tagatagattttaactgaaattatttatagggat**tcg**gatagaaggaaagaagaaaaagctgcacaa

Strain: PHX220

Allele: *syb220*

Description: Deletion of alternative *unc-68* exon 13

Wild type sequence (with deletion in double strikethrough):

agcttctccgctttctgtactttaagcacatccgattttcaatcaataatttttcaatttcaactaacttcaatttcagaagaagtctactggaact
gccacatgatgagcgacaaattgctgaagacagtatgagagacttaaacgatcgtcattctgagaaaccaaagaagggaggtcttctaa
gcaggctcagagattcaagtaatactcgcaaaaagtaagagtcattagaaaaatgtagatagattttaactgaaattatttatagcttcagg
gatagtgatagaaggaaagaagaaaaagctgcacaattgcgacaaatgaaggcaaattcaagaagttttgatgcaggaagtcttgata
cgtcaacccttccaactggtcagaaggatgtattgg~~gttagtgtttgaaagtaaaaaataaaagatgaagaatggtaaacacaatgataa~~
~~gttgattcgactcaacagcagcgcttgcaaattttaaataaaaagtctcatctacaaaactttagaacattatcacgctctccaatagtcact~~
~~agaacctttagtagtagaattattcagaagaagaaacagaagagcaggagcctatttgggtcatgcaagaccaatttcacgacgaagat~~
~~acagtgtccggtgagcagtacgccgcatcagcctctctttcagatctttcatcaacattcttaccttttccataccttttcctctgcagcatgat~~
~~actcgcatatcatactccctttctctcatacccccattacaaatctaatttctaatagaaaaacgagtctaaaattgtagcttacttcaaatttttc~~
~~ag~~cctctagcgagatgccactatccggaccaggtcgacaattgacgatcaagagaagttctattaagaaaaacaaaaaaggaaagaa
ggtatattagaaagtagagatttttcaaagtttactttttttccaggccgaaatcgctcttgagaagatggaacgagagaagaaaggctcaa
ttataccaatggatgctcagctagatgtgctccaagaaggagacgcccatgcacttgttcaccacaaggataaggttgatgaatactact
acggtatcagaatcttcccagggcaagatccatctcaagtatgggttggatgggtcaccacacaataccactattataatgtcaactttgat
ggaagtcaaggagtcagaaaatgtagattctcagaagccgatcatcacggaact

Edited sequence (left and right of the deletion with a different color and synonymous mutations are labelled in purple):

agcttctccgctttctgtactttaagcacatccgattttcaatcaataatttttcaatttcaactaacttcaatttcagaagaagtctactggaact
gccacatgatgagcgacaaattgctgaagacagtatgagagacttaaacgatcgtcattctgagaaaccaaagaagggaggtcttctaa
gcaggctcagagattcaagtaatactcgcaaaaagtaagagtcattagaaaaatgtagatagattttaactgaaattatttatagcttcagg
gatagtgatagaaggaaagaagaaaaagctgcacaattgcgacaaatgaaggcaaattcaagaagttttgatgcaggaagtcttgata
cgtcaacccttccaactggtcagaaggatgta**c**tggcct**ttg**gcgagatgccactatccggaccaggtcgacaattgacgatcaagag
aagttctattaagaaaaacaaaaaaggaaagaaggtatattagaaagtagagatttttcaaagtttactttttttccaggccgaaatcgctct
tgagaagatggaacgagagaagaaaggctcaattataccaatggatgctcagctagatgtgctccaagaaggagacgcccatgcactt
gttcaccacaaggataaggttgatgaatactactacggtatcagaatcttcccagggcaagatccatctcaagtatgggttggatgggtc
accacacaataccactattataatgtcaactttgatggaagtcaaggagtcagaaaatgtagattctcagaagccgatcatcacggaact

Strain: PHX471

Allele: *syb471*

Description: wrmScarlet (intron free) in frame upstream of *unc-68* exon 1.1

Wild type sequence:

tttgttaaaagcgctaacatgcaaatgttggatttaatgaagtaagaaagctatactataaatcccaaatctcagctcagtgaatatgctttaa
aataaaaattaactcaaatcagagcagtttttgaatttttcaactctcttcaaaaatattaaacgcacgttaaattttgtattgaaggaaatatt
attcactttactgcttgcacttgaaaatagtatttctacttttccactttaaaaaatctcaaaaacctccttggcaataaatctaaaatatccccat
aattttccgttcgttttttctgtcgtcttttgctcccgtttgttattttttttttgcgttttcccaagtggtaccagcacctctagtttttttgttttacag
atgcaaaaccagtgaaaaacggaacgaactgagtgagcagatataagaacgaaaccgaccgaacattatcagatcgacgatcgaca
acgatggccgacaaggaggagcagggaggaggcgaacaggatgatgtctcttttctccgaacggttagttttattcaaaacagacaaa
gttcaagttgacaattagttctccggttgacttttcggaggggaatatttcggctctcacttgaatgtagatcgataacatcgataacattttg
aaatattaatttctgaatttttttttgcatttaattgtgtagtacggaatgttgtacttttcaaattgtgcaaaaatgtgccacctcgcttaaacgag
tgaaaagaagaggaccctccggggtgtaagtgcgcgattcgcaaat

Edited sequence (left and right of the insertion with a different color, WormScarlet sequence is in orange and synonymous mutations are labelled in purple):

tttgttaaaagcgctaacatgcaaatgttggatttaatgaagtaagaaagctatactataaatcccaaatctcagctcagtgaatatgctttaa
aataaaaattaactcaaatcagagcagtttttgaatttttcaactctcttcaaaaatattaaacgcacgttaaattttgtattgaaggaaatatt
attcactttactgcttgcacttgaaaatagtatttctacttttccactttaaaaaatctcaaaaacctccttggcaataaatctaaaatatccccat
aattttccgttcgttttttctgtcgtcttttgctcccgtttgttattttttttttgcgttttcccaagtggtaccagcacctctagtttttttgttttacag
atgcaaaaccagtgaaaaacggaacgaactgagtgagcagatataagaacgaaaccgaccgaacattatcagatcgacgatcgaca
acgatggtcagcaagggagaggcagttatcaaggagttcatgcgtttcaaggtccacatggagggatccatgaacggacacgagttc
gagatcgagggagagggagagggacgtccatacgagggaacccaaaccgccaagctcaaggtcaccaagggaggaccactccc
attctcctgggacatcctctccccacaattcatgtacggatcccgtgccttcaccaagcacccagccgacatcccagactactacaagca
atccttcccagagggattcaagtgggagcgtgtcatgaacttcgaggacggaggagccgtcaccgtcacccaagacacctccctcga
ggacggaaccctcatctacaaggtcaagctccgtggaaccaacttcccaccagacggaccagtcatgcaaaagaagaccatgggat
gggaggcctccaccgagcgtctctacccagaggacggagtcctcaagggagacatcaagatggccctccgtctcaaggacggagg
acgttacctcgccgacttcaagaccacctacaaggccaagaagccagtccaaatgccaggagcctacaacgtcgaccgtaagctcga
catcacctcccacaacgaggactacaccgtcgtcgagcaatacgagcgttccgagggacgtcactccaccggaggaatggacgagc
tctacaagatggccgacaag**gaa**gagcagggaggaggcgaacaggatgatgtctcttttctccgaacggttagttttattcaaaacaga
caaagttcaagttgacaattagttctccggttgacttttcggaggggaatatttcggctctcacttgaatgtagatcgataacatcgataaca
ttttgaaatattaatttctgaatttttttttgcatttaattgtgtagtacggaatgttgtacttttcaaattgtgcaaaaatgtgccacctcgcttaaa
cgagtgaaaagaagaggaccctccggggtgtaagtgcgcgattcgcaaat

Strain: PHX729

Allele: *syb729*

Description: mNeonGreen in frame upstream of *unc-68* exon 1.2

Wild type sequence:

ctccttctcccgatctctcttcgttttccctcgagagacttatgtcgcccatatggcattcagccgtcccccggtcccgcctattgttttttcgt
cgctcttttttttggctcacaatttttggtgactcttcagttttcagacaaagagactccacattagaagtctgctctaggcagcaaactcata
gtattttgccaaacaagcatccgcagacaaatacacgcaaacacaattacaacgcagatataaatatattattcagtgatatgattagttag
ttccaagaaatggattttgatttcacggatatctcagatgaggatcataactatgttacaaaggtttgtttaaaagaactattctatattaaacg
gaaagtctggaaatcacagaggacagaggaaatgaatttctttatttttgataaaatttttatttccagggagacatcgtctgcctctcctgc
gttgcctcacacaatagagatggagttctcgggtcagaaaggttggtg

Edited sequence (left and right of the insertion with a different color, mNeonGreen sequence is in blue and synonymous mutations are labelled in purple):

ctccttctcccgatctctcttcgttttccctcgagagacttatgtcgcccatatggcattcagccgtcccccggtcccgcctattgttttttcgt
cgctcttttttttggctcacaatttttggtgactcttcagttttcagacaaagagactccacattagaagtctgctctaggcagcaaactcata
gtattttgccaaacaagcatccgcagacaaatacacgcaaacacaattacaacgcagatataaatatattattcagtgatatgattagttag
ttccaagaaatggtctccaagggagaggaggacaacatggcctccctcccagccacccacgagctccacatcttcggatccatcaac
ggagtcgacttcgacatggtcggacaaggaaccggaaacccaaacgacggatacgaggagctcaacctcaagtccaccaaggtaa
gtttaaacatatatatactaactaaccctgattatttaaattttcagggagacctccaattctccccatggatcctcgtcccacacatcggata
cggattccaccaatacctcccatacccagacggaatgtccccattccaagccgccatggtcgacggatccggataccaagtccaccgt
accatgcaattcgaggacggagcctccctcaccgtcaactaccgttacacctacgagggatcccacatcaaggtaagtttaaacagttc
ggtactaactaaccatacatatttaaattttcagggagaggcccaagtcaagggaaccggattcccagccgacggaccagtcatgacc
aactccctcaccgccgccgactggtgccgttccaagaagacctacccaaacgacaaggtaagtttaaacatgattttactaactaactaa
tctgatttaaattttcagaccatcatctccaccttcaagtggtcctacaccaccggaaacggaaagcgttaccgttccaccgcccgtacca
cctacaccttcgccaagccaatggccgccaactacctcaagaaccaaccaatgtacgtcttccgtaagaccgagctcaagcactccaa
gaccgagctcaacttcaaggagtggcaaaaggccttcaccgacgtcatgggaatggacgagctctacaagatggattttgatttcacgg
atatc**tca**gat**gaa**gatcataac**tac**gtt**aca**aaggtttgtttaaaagaactattctatattaaacggaaagtctggaaatcacagaggac
agaggaaatgaatttctttatttttgataaaatttttatttccagggagacatcgtctgcctctcctgcgttgcctcacacaatagagatggagt
tctcgggtcagaaaggttggtg
