# Supplementary material for: Tissue-specific isoforms of the single C. elegans Ryanodine receptor gene unc-68 control specific functions
Source: PLoS Genet. 2020 Oct 26;16(10):e1009102. doi: 10.1371/journal.pgen.1009102 (PMC7644089; doi:10.1371/journal.pgen.1009102)
Supplement: S2 File — (PDF) [file pgen.1009102.s007.pdf]

S2 File. Sequences of the *unc-68* splicing reporter minigenes

> unc-68ex13.1::mScarlet
```
ATGGGCTTCAGGGATAGTGATAGAAGGAAAGAAGAAAAAGCTGCACAATTGCGACAAATGAAGGCAAATTCAAGAAGTTTTGATGCAGGAAGTC
TTGATACGTCAACCCTTCCAACTGGTCAGAAGGATGTATTGGgttagtgtttgaaagtaaaaaataaaagatgaagaatggtaaacacaatgat
aagttgattcgactcaacagcagcgcttgcaaattttaaataaaaagtctcatctacaaaactttagaacattatcacgctctccaatagtcac
tagaacctttagtagtagaattattcagAAGAAGAAACAGAAGAGCAGGAGCCTATTTGGGaaTCATGCAAGACCAATTTCACGACGAAGATAC
AGTGTCCGgtgagcagtacgccgcatcagcctctctttcagatctttcatcaacattcttaccttttccataccttttcctctgcagcatgata
ctcgcatatcatactccctttctctcataccccccattacaaatctaatttctaatagaaaaacgagtctaaaattgtagcttacttcaaatttt
tcagCCTCTAGCGAGATGCCACTATCCGGACCAGGTCGACAATtTGACGATCAAGAGAAGTTCTATTAAGAAAAACAAAAAAGGAAAGAAGtcc
ggatccATGGTCAGCAAGGGAGAGGCAGTTATCAAGGAGTTCATGCGTTTCAAGGTCCACATGGAGGGTTCCATGAACGGACACGAGTTCGAGA
TCGAGGGAGAGGGAGAGGGACGTCCATACGAGGGAACCCAAACCGCCAAGCTCAAGGTCACCAAGgtaagtttaaacatatatatactaactaa
ccctgattatttaaattttcagGGAGGACCACTCCCATTCTCCTGGGACATCCTCTCCCCACAATTCATGTACGGTTCCCGTGCCTTCACCAAG
CACCCAGCCGACATCCCAGACTACTACAAGCAATCCTTCCCAGAGGGATTCAAGTGGGAGCGTGTCATGAACTTCGAGGACGGAGGAGCCGTCA
CCGTCACCCAAGACACCTCCCTCGAGGACGGAACCCTCATCTACAAGgtaagtttaaacagttcggtactaactaaccatacatatttaaattt
tcagGTCAAGCTCCGTGGAACCAACTTCCCACCAGACGGACCAGTCATGCAAAAGAAGACCATGGGATGGGAGGCCTCCACCGAGCGTCTCTAC
CCAGAGGACGGAGTCCTCAAGGGAGACATCAAGATGGCCCTCCGTCTCAAGGACGGAGGACGTTACCTCGCCGACTTCAAGgtaagtttaaaca
tgattttactaactaactaatctgatttaaattttcagACCACCTACAAGGCCAAGAAGCCAGTCCAAATGCCAGGAGCCTACAACGTCGACCG
TAAGCTCGACATCACCTCCCACAACGAGGACTACACCGTCGTCGAGCAATACGAGCGTTCCGAGGGACGTCACTCCACCGGAGGAATGGACGAG
CTCTACAAGactagtactTAA
```

> unc-68ex13.0::CeBFP
```
ATGGGCTTCAGGGATAGTGATAGAAGGAAAGAAGAAAAAGCTGCACAATTGCGACAAATGAAGGCAAATTCAAGAAGTTTTGATGCAGGAAGTC
TTGATACGTCAACCCTTCCAACTGGTCAGAAGGATGTATTGGgttagtgtttgaaagtaaaaaataaaagatgaagaatggtaaacacaatgat
aagttgattcgactcaacagcagcgcttgcaaattttaaataaaaagtctcatctacaaaactttagaacattatcacgctctccaatagtcac
tagaacctttagtagtagaattattcagAAGAAGAAACAGAAGAGCAGGAGCCTATTTGaGGTCATGCAAGACCAATTTCACGACGAAGATACA
GTGTCCGgtgagcagtacgccgcatcagcctctctttcagatctttcatcaacattcttaccttttccataccttttcctctgcagcatgatac
tcgcatatcatactccctttctctcataccccccattacaaatctaatttctaatagaaaaacgagtctaaaattgtagcttacttcaaattttt
cagCCTCTAGCGAGATGCCACTATCCGGACCAGGTCGACAATTGACGATCAAGAGAAGTTCTATTAAGAAAAACAAAAAAGGAAAGAAGtccgg
atccATGTCAGAGCTTATTAAGGAGAATATGCATATGAAACTGTACATGGAAGGAACCGTGGACAACCATCACTTTAAGTGCACTTCGGAGGGG
GAAGGCAAACCGTACGAGGGCACCCAGACCATGCGAATCAAGGTGGTCGAGGGAGGCCCACTGCCCTTCGCCTTCGACATTCTGgtaagtttaa
acatatatatactaactaaccctgattatttaaattttcagGCTACTAGCTTCCTGTACGGCAGCAAGACTTTTATTAATCACACCCAGGGCAT
CCCGGACTTCTTCAAACAGTCGTTCCCTGAGGGCTTCACATGGGAGCGTGTGACCACATACGAAGACGGTGGCGTTCTCACCGCTACCCAGGAC
ACCAGCCTCCAGGACGGTTGCCTCATATATAATGTGgtaagtttaaacagttcggtactaactaaccatacatatttaaattttcagAAGATCC
GGGGAGTAAACTTCACATCCAATGGTCCGGTTATGCAGAAGAAAACACTCGGCTGGGAGGCCTTCACCGAGACGCTGTATCCGGCTGACGGAGG
CCTGGAAGGTCGAAACGACATGGCCCTGAAGCTCGTCGGCGGTAGCCATCTCATCGCAAACATCAAGACCACATATAGATCCAAGAAACCGgta
agtttaaacatgattttactaactaactaatctgatttaaattttcagGCTAAGAACCTTAAAATGCCGGGAGTCTACTATGTTGACTACAGAC
TGGAACGGATCAAGGAGGCCAACAACGAGACCTACGTCGAGCAGCACGAGGTTGCAGTTGCCAGATACTGCGACCTCCCGAGCAAACTGGGTCA
CAAGCTTAATactagtactTAA
```

> unc-68ex13::ScarBFP
```
ATGGGCTTCAGGGATAGTGATAGAAGGAAAGAAGAAAAAGCTGCACAATTGCGACAAATGAAGGCAAATTCAAGAAGTTTTGATGCAGGAAGTC
TTGATACGTCAACCCTTCCAACTGGTCAGAAGGATGTATTGGgttagtgtttgaaagtaaaaaataaaagatgaagaatggtaaacacaatgat
aagttgattcgactcaacagcagcgcttgcaaattttaaataaaaagtctcatctacaaaactttagaacattatcacgctctccaatagtcac
tagaacctttagtagtagaattattcagAAGAAGAAACAGAAGAGCAGGAGCCTATTTGGGaaTCATGCAAGACCAATTTCACGACGAAGATAC
AGTGTCCGgtgagcagtacgccgcatcagcctctctttcagatctttcatcaacattcttaccttttccataccttttcctctgcagcatgata
ctcgcatatcatactccctttctctcataccccccattacaaatctaatttctaatagaaaaacgagtctaaaattgtagcttacttcaaatttt
tcagCCTCTAGCGAGATGCCACTATCCGGACCAGGTCGACAATtTGACGATCAAGAGAAGTTCTATcAAGAAAAACAAAAAAGGAAAGAAGtcc
ggatccATGGTCAGCAAGGGAGAGGCAGTTATCAAGGAGTTCATGCGTTTCAAGGTCCACATGGAGGGTTCCATGAACGGACACGAGTTCGAGA
TCGAGGGAGAGGGAGAGGGACGTCCATACGAGGGAACCCAAACCGCCAAGCTCAAGGTCACCAAGgtaagtttaaacatatatatactaactaa
ccctgattatttaaattttcagGGAGGACCACTCCCATTCTCCTGGGACATCCTCTCCCCACAATTCATGTACGGTTCCCGTGCCTTCACCAAG
CACCCAGCCGACATCCCAGACTACTACAAGCAATCCTTCCCAGAGGGATTCAAGTGGGAGCGTGTCATGAACTTCGAGGACGGAGGAGCCGTCA
CCGTCACCCAAGACACCTCCCTCGAGGACGGAACCCTCATCTACAAGgtaagtttaaacagttcggtactaactaaccatacatatttaaattt
tcagGTCAAGCTCCGTGGAACCAACTTCCCACCAGACGGACCAGTCATGCAAAAGAAGACCATGGGATGGGAGGCCTCCACCGAGCGTCTCTAC
CCAGAGGACGGAGTCCTCAAGGGAGACATCAAGATGGCCCTCCGTCTCAAGGACGGAGGACGTTACCTCGCCGACTTCAAGACCACCTACAAGG
CCAAGAAGCCAGTCCAAATGCCAGGAGCCTACAACGTCGACCGaAAGCTCGACATCACCTCCCACAACGAGGACTACACCGTCGTCGAGCAATA
CGAGCGTTCCGAGGGACGTCACTCCACCGGAGGAATGGACGAGCTCTACAAGaactagtactTATGTCAGAGCTTATTAAGGAGAATATGCATA
TGAAACTGTACATGGAAGGAACCGTGGACAACCATCACTTTAAGTGCACTTCGGAGGGGGAAGGCAAACCGTACGAGGGCACCCAGACCATGCG
AATCAAGGTGGTCGAGGGAGGCCCACTGCCCTTCGCCTTCGACATTCTGGCTACTAGCTTCCTGTACGGCAGCAAGACTTTTATTAATCACACC
CAGGGCATCCCGGACTTCTTCAAACAGTCGTTCCCTGAGGGCTTCACATGGGAGCGTGTGACCACATACGAAGACGGTGGCGTTCTCACCGCTA
CCCAGGACACCAGCCTCCAGGACGGTTGCCTCATATATAATGTGAAGATCCGGGGAGTAAACTTCACATCCAATGGTCCGGTTATGCAGAAGAA
AACACTCGGCTGGGAGGCCTTCACCGAGACGCTGTATCCGGCTGACGGAGGCCTGGAAGGTCGAAACGACATGGCCCTGAAGCTCGTCGGCGGT
AGCCATCTCATCGCAAACATCAAGACCACATATAGATCCAAGAAACCGGCTAAGAACCTTAAAATGCCGGGAGTCTACTATGTTGACTACAGAC
TGGAACGGATCAAGGAGGCCAACAACGAGACCTACGTCGAGCAGCACGAGGTTGCAGTTGCCAGATACTGCGACCTCCCGAGCAAACTGGGTCA
CAAGCTTAATTAA
```
