# Supplementary material for: Tissue-specific isoforms of the single C. elegans Ryanodine receptor gene unc-68 control specific functions
Source: PLoS Genet. 2020 Oct 26;16(10):e1009102. doi: 10.1371/journal.pgen.1009102 (PMC7644089; doi:10.1371/journal.pgen.1009102)
Supplement: S3 File — (PDF) [file pgen.1009102.s008.pdf]

S3 File. Raw data used for tissue-specific SRT analysis

**Replicate name in Ma *et al.***	**Total number of transcripts analyzed**	**Number of ex1.1-containing transcripts (flagged as *trans*-site, *position V-6929038,* in Ma *et al.*)**	**Number of ex1.2-containing transcripts (flagged as *cis*-site, *position V-6924806,* in Ma *et al.*)**
muscle_G16C_d12_1	571844	0	0
muscle_G16C_d12_2	2232792	112	0
neuron_G16C_d12_1	164667	0	5
neuron_G16C_d12_2	317345	2	9

Raw data were retrieved in the supplements from: Ma, X., Zhan, G., Sleumer, M. C., Chen, S., Liu, W., Zhang, M. Q., & Liu, X. (2016). Analysis of C. elegans muscle transcriptome using trans-splicing-based RNA tagging (SRT). Nucleic acids research, 44(21), e156. https://doi.org/10.1093/nar/gkw734
